# Modeling of soil moisture movement and wetting behavior under point-source trickle irrigation

**DOI:** 10.1038/s41598-023-41435-4

**Published:** 2023-09-11

**Authors:** Dinesh Kumar Vishwakarma, Rohitashw Kumar, Salwan Ali Abed, Nadhir Al-Ansari, Amit Kumar, Nand Lal Kushwaha, Devideen Yadav, Anita Kumawat, Alban Kuriqi, Abed Alataway, Ahmed Z. Dewidar, Mohamed A. Mattar

**Affiliations:** 1 Department of Irrigation and Drainage Engineering, Govind Ballabh Pant University of Agriculture and Technology, Pantnagar, Uttarakhand 263145 India; 2 College of Agricultural Engineering and Technology, Sher-e-Kashmir University of Agricultural Sciences and Technology of Kashmir, Shalimar Campus, Srinagar, Jammu and Kashmir 190025 India; 3 College of Science, University of Al-Qadisiyah, Qadisiyyah, 58002 Iraq; 4 Department of Civil, Environmental, and Natural Resources Engineering, Lulea University of Technology, 97187 Lulea, Sweden; 5 Division of Fruit Science, Faculty of Horticulture, Sher-e-Kashmir University of Agricultural Sciences and Technology of Kashmir, Shalimar Campus, Srinagar, Jammu and Kashmir 190025 India; 6 Division of Agricultural Engineering, ICAR-Indian Agricultural Research Institute, New Delhi, 110012 India; 7 Division of Soil Science and Agronomy, ICAR-Indian Institute of Soil and Water Conservation, Dehradun, India; 8 ICAR-Indian Institute of Soil and Water Conservation, Research Centre, Kota, 324002 Rajasthan India; 9 CERIS, Instituto Superior Técnico, University of Lisbon, 1649-004 Lisbon, Portugal; 10 Civil Engineering Department, University for Business and Technology, Pristina, Kosovo; 11 Prince Sultan Bin Abdulaziz International Prize for Water Chair, Prince Sultan Institute for Environmental, Water and Desert Research, King Saud University, Riyadh, 11451 Saudi Arabia; 12 Department of Agricultural Engineering, College of Food and Agriculture Sciences, King Saud University, Riyadh, 11451 Saudi Arabia; 13 Agricultural Engineering Research Institute (AEnRI), Agricultural Research Centre, Giza, 12618 Egypt

**Keywords:** Software, Civil engineering

## Abstract

The design and selection of ideal emitter discharge rates can be aided by accurate information regarding the wetted soil pattern under surface drip irrigation. The current field investigation was conducted in an apple orchard in SKUAST- Kashmir, Jammu and Kashmir, a Union Territory of India, during 2017–2019. The objective of the experiment was to examine the movement of moisture over time and assess the extent of wetting in both horizontal and vertical directions under point source drip irrigation with discharge rates of 2, 4, and 8 L h^−1^. At 30, 60, and 120 min since the beginning of irrigation, a soil pit was dug across the length of the wetted area on the surface in order to measure the wetting pattern. For measuring the soil moisture movement and wetted soil width and depth, three replicas of soil samples were collected according to the treatment and the average value were considered. As a result, 54 different experiments were conducted, resulting in the digging of pits [3 emitter discharge rates × 3 application times × 3 replications × 2 (after application and 24 after application)]. This study utilized the Drip-Irriwater model to evaluate and validate the accuracy of predictions of wetting fronts and soil moisture dynamics in both orientations. Results showed that the modeled values were very close to the actual field values, with a mean absolute error of 0.018, a mean bias error of 0.0005, a mean absolute percentage error of 7.3, a root mean square error of 0.023, a Pearson coefficient of 0.951, a coefficient of correlation of 0.918, and a Nash–Sutcliffe model efficiency coefficient of 0.887. The wetted width just after irrigation was measured at 14.65, 16.65, and 20.62 cm; 16.20, 20.25, and 23.90 cm; and 20.00, 24.50, and 28.81 cm in 2, 4, and 8 L h^−1^_,_ at 30, 60, and 120 min, respectively, while the wetted depth was observed 13.10, 16.20, and 20.44 cm; 15.10, 21.50, and 26.00 cm; 19.40, 25.00, and 31.00 cm_,_ respectively. As the flow rate from the emitter increased, the amount of moisture dissemination grew (both immediately and 24 h after irrigation). The soil moisture contents were observed 0.4300, 0.3808, 0.2298, 0.1604, and 0.1600 cm^3^ cm^−3^ just after irrigation in 2 L h^−1^ while 0.4300, 0.3841, 0.2385, 0.1607, and 0.1600 cm^3^ cm^−3^ were in 4 L h^−1^ and 0.4300, 0.3852, 0.2417, 0.1608, and 0.1600 cm^3^ cm^−3^ were in 8 L h^−1^ at 5, 10, 15, 20, and 25 cm soil depth in 30 min of application time. Similar distinct increments were found in 60, and 120 min of irrigation. The findings suggest that this simple model, which only requires soil, irrigation, and simulation parameters, is a valuable and practical tool for irrigation design. It provides information on soil wetting patterns and soil moisture distribution under a single emitter, which is important for effectively planning and designing a drip irrigation system. Investigating soil wetting patterns and moisture redistribution in the soil profile under point source drip irrigation helps promote efficient planning and design of a drip irrigation system.

## Introduction

Water is essential for the survival of human beings, plants, and animals, and it is a significant agricultural input. Water scarcity is a terrible reality in many regions of the world due to ever-growing population pressure; however, many locations have little rainfall, and crop/fruit production relies heavily on irrigation. In contrast to the massive amounts of water utilized in agriculture, more efficient irrigation practices are promoted^1^. Soil moisture is crucial in agriculture, affecting plant growth and crop yield^2–5^. Irrigation systems play a crucial role in maintaining optimal soil moisture levels. One of the most effective techniques is drip irrigation, which provides water directly to the root zone of plants ^6–10^. To optimize the design and management of drip irrigation systems, it is important to understand the movement of soil moisture under different conditions^11–15^.

Drip irrigation is often used because it allows for low-rate irrigation, has high water efficiency, and is easily automated. Traditional irrigation systems, such as flood and border irrigation, waste much water and are inefficient, which is bad for the natural economy and long-term growth^16^. Drip irrigation effectively distributes water and nutrients directly in the plant rhizosphere, maximizing irrigation water efficiency and reducing root zone chemical leaching. To improve irrigation efficiency, proper design and planning are crucial. Key design elements, such as lateral spacing, operating pressure, emitter spacing, emitter point position, and emitter discharge rates, must be established and maintained, including proper irrigation scheduling and a sound fertigation strategy^3, 9^. The supply of water and fertilizer in the root zone must align with crop requirements to ensure optimal effectiveness^4, 17–21^. However, proper design and planning of the system can further enhance irrigation efficiency. In order to build an efficient system, various design features must be properly built (lateral spacing, operating pressure, emitter spacing, proper position of emitter point, emitter discharge rates) and maintained (e.g., irrigation scheduling, fertigation strategy)^3, 9, 10^. The rates and locations of water and fertilizer supplies in the root zone must match crop requirements^3, 22–24^.

To maximize water efficiency, the drip irrigation system is built to fit the soil and plant parameters, such as rooting depth, crop water requirements, irrigation scheduling, and application rate. Many variables contributed to the increased yield of drip irrigation: (a) better soil water distribution, (b) more precise water and fertilizer application, (c) soil water distribution according to the root zone of the crops, and (d) little water loss through evaporation from the soil surface^8, 25^. Only two soil water distribution parameters, wetted soil radius (horizontal water movement) and wetted soil depth (vertical water movement), were considered as primary indicators relating moisture distribution concerning root zone area from a drip emitter on the soil surface in drip irrigation system design^26–28^. Exact data on the axis of horizontal and vertical soil water movement from a point source drip emitter can aid in determining the root zone management depth, irrigation quantities, and frequency^29, 30^.

Drip system design criteria are based on soil physical and hydraulic characteristics^31^. The wetting dimension of soil single under point source drip irrigation should be well-defined to ensure appropriate matching between these wetted soil pattern dimensions and the distance between emitters and laterals, as well as the rooting depth ^32^. Several researchers emphasized the wetted dimension/shape of wetted soil, as well as the wetted bulb volume and the spatial soil moisture distribution under point source drip irrigation, which are heavily influenced by soil hydraulic characteristics (soil texture, saturated hydraulic conductivity, bulk density, and initial moisture content), emitter discharge rates, number of emitters, emitter spacing, and their interactions^8, 9, 33–46^. Field planners will benefit more from accurate data on the horizontal and vertical wetting front advance of the soil in order to design better row and plant emitter discharge and emitter spacing, as well as a design operating pressure, in order to reduce costs and increase water use efficiency, effectiveness, and productivity^35–37, 45, 47–49^. Field observations are expensive, and it might not be easy to visualize and distinguish wetted soil measurements from dry soil dimensions. An option might be to use analytical, numerical, empirical, or physical models can be used to calculate wetted soil width and depth^45^. Many studies have focused on the wetted soil dimension and produced analytical models for the simulation of the wetted soil width and the vertical depth of the wetted soil dimensions using the physical approach of the governing flow equation^43, 44, 50, 51^. The wetted soil width and depth could be forecasted using numerical models^41, 47, 52–54^. Data from field and lab tests and regression analysis were used to create many empirical models. These models incorporate empirical calculations to predict the wetted soil width and depth of the wet bulb as a function of soil physical and hydraulic phenomena and emitter discharge rate^28, 35, 55–59^.

There have been numerous studies on modeling soil moisture movement using different simulation models. Arraes et al.^60^ developed a numerical model to simulate water distribution as well as the shape of wetted soil volume created by a point source irrigation system close to the surface (dripper) based on soil hydraulic properties and irrigation system parameters. The proposed numerical model proved to be extremely accurate as compared to other numerical models reported in the literature and in comparison to the model developed from the measured data as well as with other results reported in the literature. When changing elements like the drip tube's installation depth, the distance between emitters, and the soil moisture levels, the applicability is seen. Jamei et al*.*^12^'s laboratory analysis for wetting radius was the subject of another study. Different recurrent neural network (RNN) based models were developed and evaluated. Result revealed that bidirectional recurrent neural network (Bi-RNN) showed higher accuracy. In this regard, Rocha et al.^13^ looked at how well a 2D numerical model represented the distribution of water and the development of wet bulbs resulting from a subsurface irrigation system. They also verified the accuracy of the model. The findings indicated that the model can be effectively employed in designing and managing subsurface irrigation systems, offering favorable outcomes for different simulated scenarios. Sun et al.^14^ simulated the soil water movement and root water uptake using HYDRUS-2D in northeast China for greenhouse tomatoes. The performance of the HYDRUS-2D model in simulating soil water movement was successful, as indicated by the evaluation metrics. The root mean square error (RMSE) values ranged from 0.014 to 0.027, the mean relative error (MRE) values ranged from 0.062 to 0.126, and the coefficient of determination (R^2^) values ranged from 79 to 92%. These results were obtained by comparing the model simulations with two-year field measurements. To investigate how soil texture, initial moisture content, moistube specific discharge, and irrigation time affect the moisture distribution of a soil wetting body, Fan et al.^15^ also used numerical simulations with HYDRUS-2D. The simulation results showed that the soil moisture content peaks at the moistube location, and that the soil texture and the moistube-specific discharge have a significant impact on this value.

However, the use of Drip-Irriwater software has received limited attention in the literature^47^. Drip-Irriwater is a software that simulates soil moisture movement under different irrigation conditions, including point source drip irrigation. The software is based on the Finite Difference Method. It considers various factors that affect soil moisture movement, such as soil type, plant type, and irrigation rate. Drip-Irriwater code, a new and eco-friendly interface (software, v. 1.0), was utilized to estimate the wetting pattern in harmonized or stratified soils under a single-point source of surface drip irrigation^47^. The Drip-Irriwater results include the width and depth of wetted soil dimensions and soil moisture redistributions. Arbat et al.^47^ statistically compared results obtained with HYDRUS and filed test field tests conducted on three different types of soil and found that Drip-Irriwater had a superior forecasting feature to HYDRUS and the results of field tests (RMSE = 0.007 cm^3^ cm^−3^ and R^2^ = 0.931 for soil water content at vertically and RMSE = 0.013 cm^3^ cm^−3^ & R^2^ = 0.934 for soil water content at horizontally). Thus, Drip-Irriwater could be used for forecasting wetted soil dimensions and soil moisture distribution around drip emitters. A few studies have evaluated the accuracy of Drip-Irriwater in predicting soil moisture movement. These studies have shown that Drip-Irriwater provides accurate and reliable results, especially for simulating soil moisture movement under point source drip irrigation. The software is a useful tool for irrigation design and management, as it provides information on soil wetting patterns and soil moisture distribution, which is crucial for effectively planning and designing a drip irrigation system.

To ensure a successful point source drip irrigation system, the characteristics of plant root distribution must be matched with the wetting front and soil moisture distribution during the design phase. The appropriate distance between laterals, the proper distance between emitters, and the right depth at which emitters should be positioned all depend on an understanding of the wetting pattern horizontally and vertically. Adopting the consideration, it could be possible to increase crop yield and quality while also using water and plant nutrients better, resulting in more precise and effective management. The literature review suggests a need for further research on using Drip-Irriwater software for modeling soil moisture movement, especially in different soil and climate conditions.

 The goal of this study is to introduce Drip-Irriwater, a brand-new, user-friendly software program that determines how soil is moistened during surface drip irrigation in uniform or layered soils. This study aims to add to the body of knowledge by offering a thorough analysis of how well Drip-Irriwater predicts soil moisture movement under various conditions. Additionally, the validation of Drip-Irriwater is presented, comparing the results obtained with the results of field tests that were conducted in real field conditions. Also, the approximate estimate and field observation of wetting front dimension and estimate the soil water dynamics/distribution after end of irrigation and 24 h after the end of irrigation were compared in loam soil under a point source of trickle irrigation.

## Methodology

### Study area

**Figure 1:**
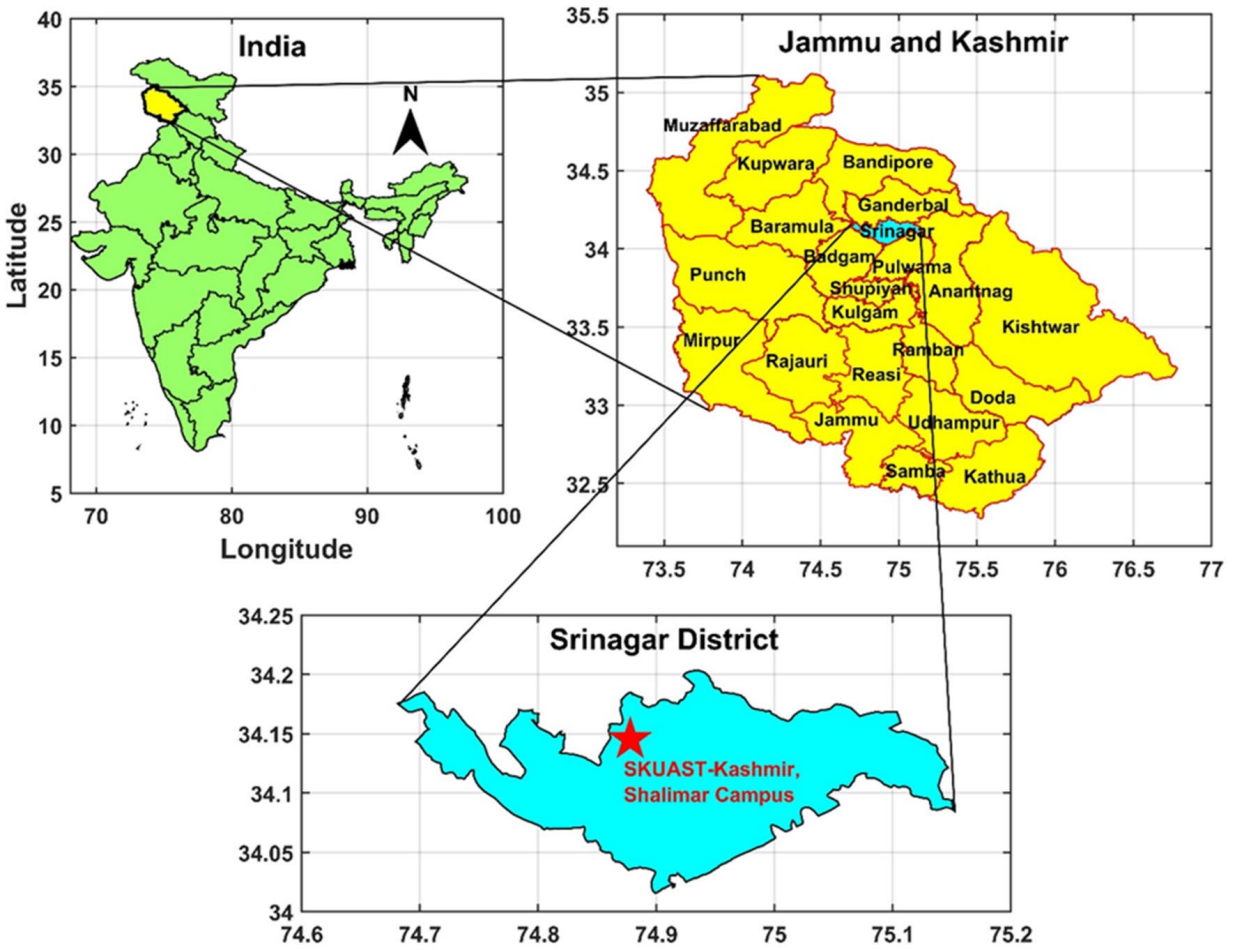
Study area (made by arcGIS 10.2 version).

The apple orchard experimental field at the Sher-e-Kashmir University of Agricultural Sciences and Technology of Kashmir's College of Agricultural Engineering and Technology in Srinagar, which is located at an elevation of 1606 meters above mean sea level, was used for this study. It was located at 34.14° N latitude and 74.87° E longitude (Fig. 1). The experimental site's soil was deep and well-drained, with two layers of loam up to 30 cm deep and sandy loam soil with an average of 42.5% silt, 47.6% fine sand, 1.9% coarse sand, and 8.5% clay (Table 1).

**Table 1:** Physical characteristics of different soil layers from experimental field plots used in the investigation.

Soil type	Soil horizon	Depth (cm)	Clay (%)	Silt (%)	Fine sand (%)	Coarse sand (%)	Bulk density (g cm^−3^)	Porosity (%)	pH	EC (dSm^−1^)
Loam	A	0–15	10	40	47.72	2.28	1.33	44	5.36	0.06
Loam	B	15–30	8	46	43.82	2.18	1.52	44	6.05	0.06
Sandy loam	C	30–45	6	46	46.71	1.29	1.61	37.5	6.54	0.09
Sandy loam	D	45–60	10	38	52.05	1.95	1.62	37.5	6.45	0.08
Average: loam	(Up to 60 cm depth)		8.5	42.5	47.57	1.925	1.52	40.75	6.10	0.073

### Soil physical-hydraulic assets of the investigation field

The four layers that comprise the natural soil profile range in depth from 0 to 15 cm, 15 to 30 cm, 30 to 45 cm, and 45 to 60 cm. The physical characteristics of different layers of soil are summarized in Table 1. The soil-hydraulic property of the field plot was assessed using the RETC program (an open source program), which used soil layers ranging from 0 to 60 cm depth^61–64^, is illustrated in Table 2. Based on the experimental data of water retention curve and hydraulic conductivity derived from the outflow data in one step, the fitting parameters of the widespread Mualem-van Genuchten model^61, 65^ were calculated using the RETC software (van Genuchten et al.^62^). When one observed conductivity value (not necessarily at saturation) is available, the RETC program can be used to predict the hydraulic conductivity from observed soil water retention data. Analytical functions can also be fitted simultaneously to observed water retention and hydraulic conductivity data using the program. A variety of program options are illustrated with a number of examples. A user manual is included with the program, in which there are detailed instructions on how to use the computer program along with examples of files for input and output files, as well as instructions on how to prepare the data input.

**Table 2:** Soil hydraulic property parameters of the van G-M equations for the four-soil profile (0–60 cm) used in the experiments.

Soil type	Soil horizon	Depth (cm)	Ѳ_r_ (cm^3^ cm^−3^)	Ѳ_s_ (cm^3^ cm^−3^)	α (cm^−1^)	n	m	K_sat_ (cm min^−1^)
Loam	A	0–15	0.078	0.43	0.036	1.56	0.359	0.0173
Loam	B	15–30	0.078	0.43	0.036	1.56	0.359	0.0173
Sandy loam	C	30–45	0.065	0.41	0.075	1.89	0.4709	0.0737
Sandy loam	D	45–60	0.065	0.41	0.075	1.89	0.4709	0.0737

A crucial aspect of soil hydraulics is the soil water retention curves (SWRC), which are defined as the soil water content as a function of the soil matric potential (Ψ_m_). Using the computer program, a retention model based on Van Genuchten was used to determine the experimental field plot's soil water retention curve (SWRC)^61^.

1 $${\theta }_{\Psi }= {\theta }_{r}+\frac{\left({\theta }_{s}-{\theta }_{r}\right)}{{\left[1+{\left(\alpha \Psi \right)}^{n}\right]}^{m}}$$

**Figure 2:**
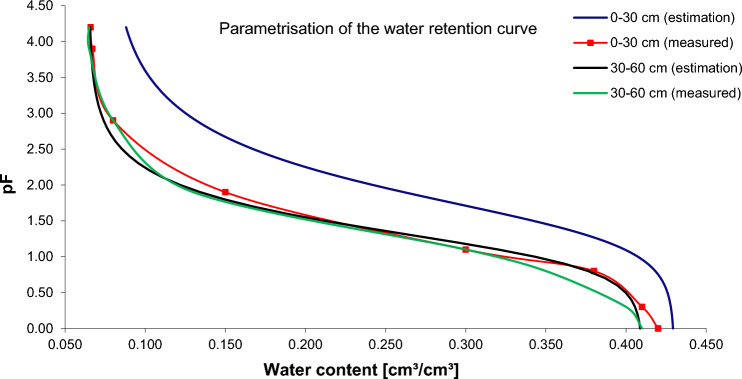
SWRC for the sandy loam soil in the investigated site.

The volumetric soil moisture content at a given soil–water potential, Ψ, can be represented by ${\theta }_{\Psi }$. ${\theta }_{r}$ and ${\theta }_{s}$ represent the residual and saturated volumetric moisture content, respectively. On the basis of field data, RETC software was used to determine the unknown parameters of the Mualem-van Genuchten (M-vG) model in the parameter optimization process to fit the water retention and unsaturated hydraulic conductivity functions (θ_r_, α and n). The inverse of air entry suction, α, and constants n and m, which are related to the pore-size distribution (m = 1 − 1/n), are used in the Van Genuchten equation to fit the soil moisture retention curve. The curve for sandy soil after putting parameters into the Van Genuchten equation is shown in Fig. 2. The input data for the iterator is the pF-value, the measured parameter for the pF-Meter. The soil matric potential is logarithmically equivalent to the absolute value of soil matric potential^66^. The formula for converting pF to pΨ and vice-versa is show in Eqs. (2) and (3) ^66–68^:

2 $$\Psi = {10}^{\mathrm{pF}}$$

3 $$\mathrm{pF}\hspace{0.17em}=\hspace{0.17em}\mathrm{log\Psi }$$

where ψ is the Matric potential in cm. Soil water retention curves (SWRC) were plot using the soil water content as a function soil matric potential in Microsoft Excel 2019.

### Experimental field layout and treatment

**Figure 3:**
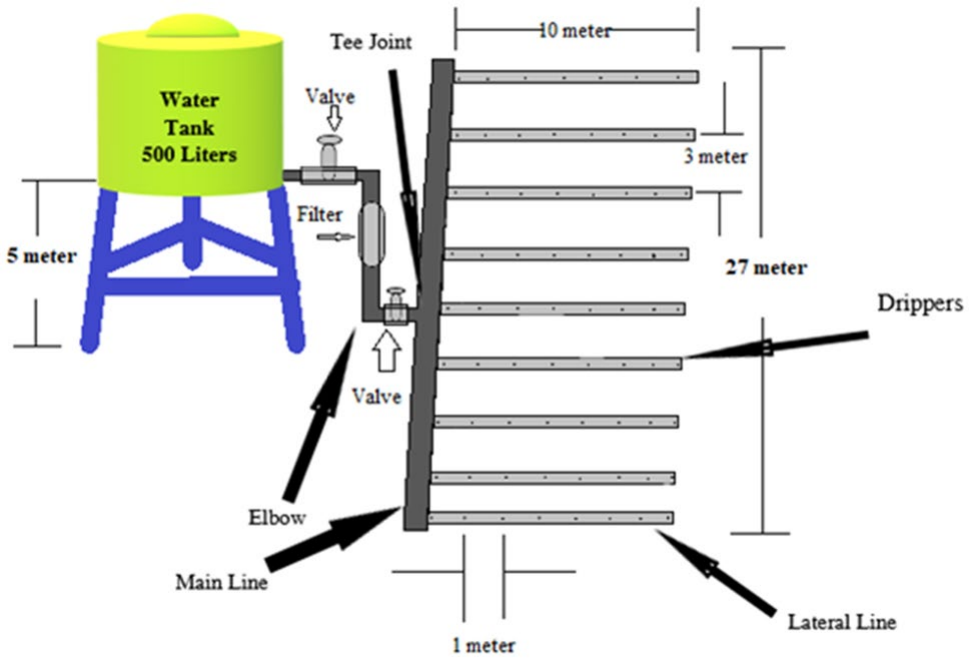
Gravity-fed drip irrigation systems were installed in the experimental field plot.

**Figure 4:**
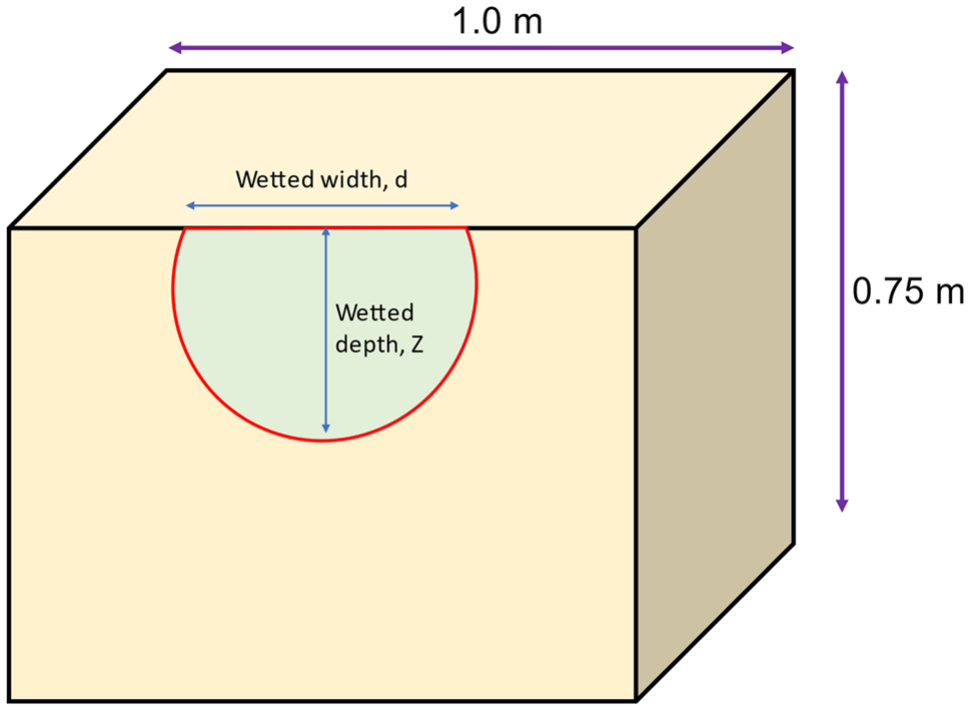
Sketch of the vertically cut of soil used for the experiments. (d: wetting width, Z: wetting depth).

A gravity-fed drip irrigation system was installed at the experimental site (Fig. 3). The water tank in the reservoir was built on an iron foundation. It includes, among other things, a 500-L water tank, a 50-mm main pipeline, a 12-mm laterals line, a pressure gauge, a sand spectator, a bypass system, two valves, and drip emitters, as well as a 50-mm main pipeline. To maintain the exact emitter discharge, the water level in the reservoir was measured every 20 min. After each irrigation, a hole was drilled around 1.0 m wide and 0.75 m deep. The width and depth of wetted soil and soil water distribution along each axis were measured (Fig. 4). Infiltration from a single emitter was used in each experiment, which was carried out at a separate position on the same experimental field plot. A varied irrigation volume was employed for each irrigation experiment. The studies evaluate how variances in soil moisture distribution altered as the soil depth varied as a function of two factors: emitter discharge rate, irrigation time duration, and measuring periods after irrigation (just after and 24 h after). Therefore, three different emitters were used (2, 4, and 8 L h^−1^), and three irrigation time durations of 30, −60, and −120 min were used. The wetted soil width and depth were measured after 30-, 60-, and −120 min just after irrigation and 24 h after irrigation applied. For measuring the soil moisture movement and wetted soil width and depth, three replica of soil sample were collected according the treatment and the average value were considered. This resulted in a total of 54 separate experiments and pits dug [3 emitter discharge rates × 3 application times × 3 replications × 2 (after application and 24 after application)]. For each time interval for a selected emitter type, three replications (pits) were dug into the undisturbed soil, and the wetted face and soil moisture of soil at each site in each pit was measured vertically and horizontally and the average value was calculated based on the vertical and horizontal wetting distances recorded in the grid.

**Figure 5:**
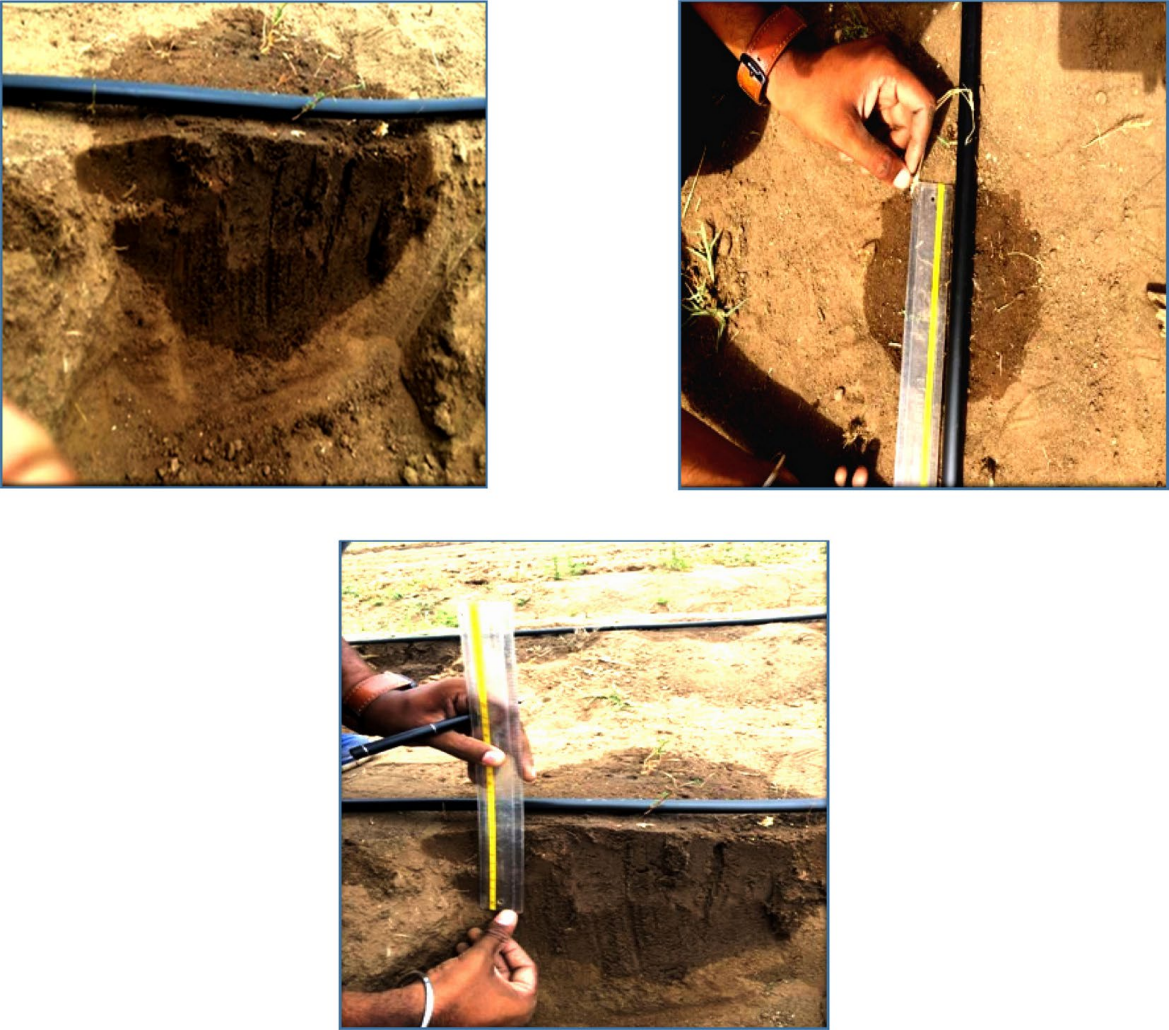
Measurement of wetting patterns in drip irrigation at the end of irrigation.

Kashmir is India's largest apple grower and main fruit crop of J&K State. Most famous variety is Red Delicious, generally, this variety required 1 × 3 spacing. Thus, the emitter spacing were chosen as per required plant spacing. 2, 4 and 8 L h^−1^ are the most common type emitter (2 L h^−1^ when planting is done in clay soils; 4 to 8 L h^−1^ when planting is done in sandy soils; and 2 to 4 L h^−1^ when planting is done in loamy soils)^69^. In this research, half layer of soil was loam and half were sandy loam, Thus, both three type emitters were selected for this experiment. Irrigation duration were based crop water requirement^70^ to observed the moisture movement and wetted soil width and depth. Table 3 depicts a variety of emitter discharge rates, irrigation times, and inter-emitter distances. Field observations were used to determine soil water content and wetting pattern dimensions, which were then calculated using Drip-Irriwater at horizontal distances of 5, 10, 15, 20, 25, 30, 35, 40, 45, 50, 55, and 60 cm and vertical depths of 5, 10, 15, 20, 25, 30, 35, 40, 45, 50, 55, and 60 cm from the point source (emitter) (Fig. 5). All parameters were visually compared with Drip-Irriwater model. At the end of irrigation and 24 h later, the maximum wetted soil width and depth and soil water contents measured and the predicted value of maximum wetted soil width and depth and soil water contents were compared. The initial soil moisture content of the experimental plot was 0.16 cm^3^ cm^−3^.

**Table 3:** Combinations of emitter discharge rate, emitter arrangement, and irrigation duration.

Emitter Discharge rate (L h^−1^)	Dripper layout (m)	Irrigation time duration (min)
2	1 × 3	30
4	1 × 3	60
8	1 × 3	120
2	1 × 3	30
4	1 × 3	60
8	1 × 3	120
2	1 × 3	30
4	1 × 3	60
8	1 × 3	120

### Drip-Irriwater

Drip Irrigation-Water Software is a computer program designed to help farmers and irrigation managers optimize water use in their fields. It is a specialized software specifically designed for drip irrigation systems, widely used in agriculture due to their efficiency and effectiveness in water use. The software provides various features and tools to help users manage their irrigation systems, including scheduling and controlling water flow to crops, monitoring soil moisture levels, and tracking water usage^47^. The software can be integrated with sensors and other devices in the field to provide real-time data and information about the irrigation system. This data can then be used to make informed decisions about water use and optimize the system's performance.

One of the key benefits of Drip Irrigation-Water Software is the ability to conserve water and reduce waste. The software can help farmers use just the right amount of water for optimal growth and yield by monitoring soil moisture levels and controlling water flow to crops. This conserves water and can reduce the risk of over-irrigation, which can lead to waterlogging and other problems. Another benefit of Drip Irrigation-Water Software is the ability to improve the efficiency of irrigation systems. The software can help farmers schedule water applications at the right time and rate, reducing the risk of runoff and ensuring that water is applied where needed. This can lead to increased productivity and yield and improved water use efficiency. Drip Irrigation-Water Software is essential for farmers and irrigation managers looking to optimize water use in their fields. It provides a range of features and tools to help manage irrigation systems and make informed water-use decisions, leading to improved efficiency and reduced waste.

Richards' equation is a main tool for studying soil water infiltration and redistribution when a drip source is involved^71^. Assuming there is symmetry around the vertical axis that passes through the emitter, the equation can be formulated as follows:

4 $$\frac{\partial h}{\partial t}= \frac{\partial }{\partial x}\left(K\frac{\partial h}{\partial x}\right)+\frac{\partial }{\partial z}\left(K\frac{\partial H}{\partial z}\right)$$

where, the variable "h" denotes the volumetric soil water content, “t” represents time, and "x" and "z" are the corresponding horizontal and vertical coordinates, respectively. The term "H" symbolizes the hydraulic head, and "K" stands for the hydraulic conductivity function, which depends on the value of "h".

The transformation of the differential equation (Eq. 4) results in a set of algebraic functions. These functions correspond to the variables at a specific position and time, and their values are influenced by their respective locations within the flow domain. To address Eq. (4) effectively, drip Irri-water model follow a specific procedure, which is outlined below:

The flow rate "Q" (expressed in L^3^ T^−1^) between two consecutive cells is determined using the following formula:

5 $$Q=q\times A$$

where q is the water flow density and A the surface area of the water, flowing from one cell to a consecutive one. The flow rate "Q" is computed using Buckingham–Darcy's law, taking into account the hydraulic conductivity "K(h)" corresponding to a specific water content value. Additionally, it considers "∆H," the difference in hydraulic potential between two consecutive cells, where "H" is defined as negative when the soil is unsaturated. Furthermore, "∆L" represents the distance between these two cells. The formula is expressed as follows:

6 $$Q=-K\left(\theta \right)\frac{\partial H}{\partial L}\times A$$

The change in volumetric soil water content in the ring corresponding to the generic cell (i, j) during a time step (Δ*t*) is given by:

7 $${\Delta \theta }_{i,j}=\frac{{\Delta Q}_{i,j}}{{V}_{i,j}}\times \Delta t$$

where V_ij_ represents the volume of the ring associated with cell (i, j). Thus, the soil water content change (${\Delta \theta }_{i,j}$) is calculated as:

8 $${\Delta \theta }_{i,j}=\frac{{\Delta Q}_{i,j}}{\pi \left({R}_{j+1}^{2}-{R}_{j}^{2}\right){\Delta }_{i}}\times \Delta t$$

where R_j_ and R_j+1_ denote the radii of the rings, and ${\Delta }_{i}$ represents the thickness of the cell in the vertical direction. The volumetric soil water content at ring (i, j) for the next time step (t + $\Delta t$) is then given by:

9 $${\theta }_{t+\Delta t}={\theta }_{t}+{\Delta \theta }_{i,j}$$

Starting with the cell (1, 1) closest to the emitter and moving up to the cell (i_max_, j_max_), these calculations are done for every cell in the flow domain. The water contents for each cell are updated, and this procedure is repeated for every time step ($\Delta t$) until the final specified time in the simulation is reached^47^.

**Figure 6:**
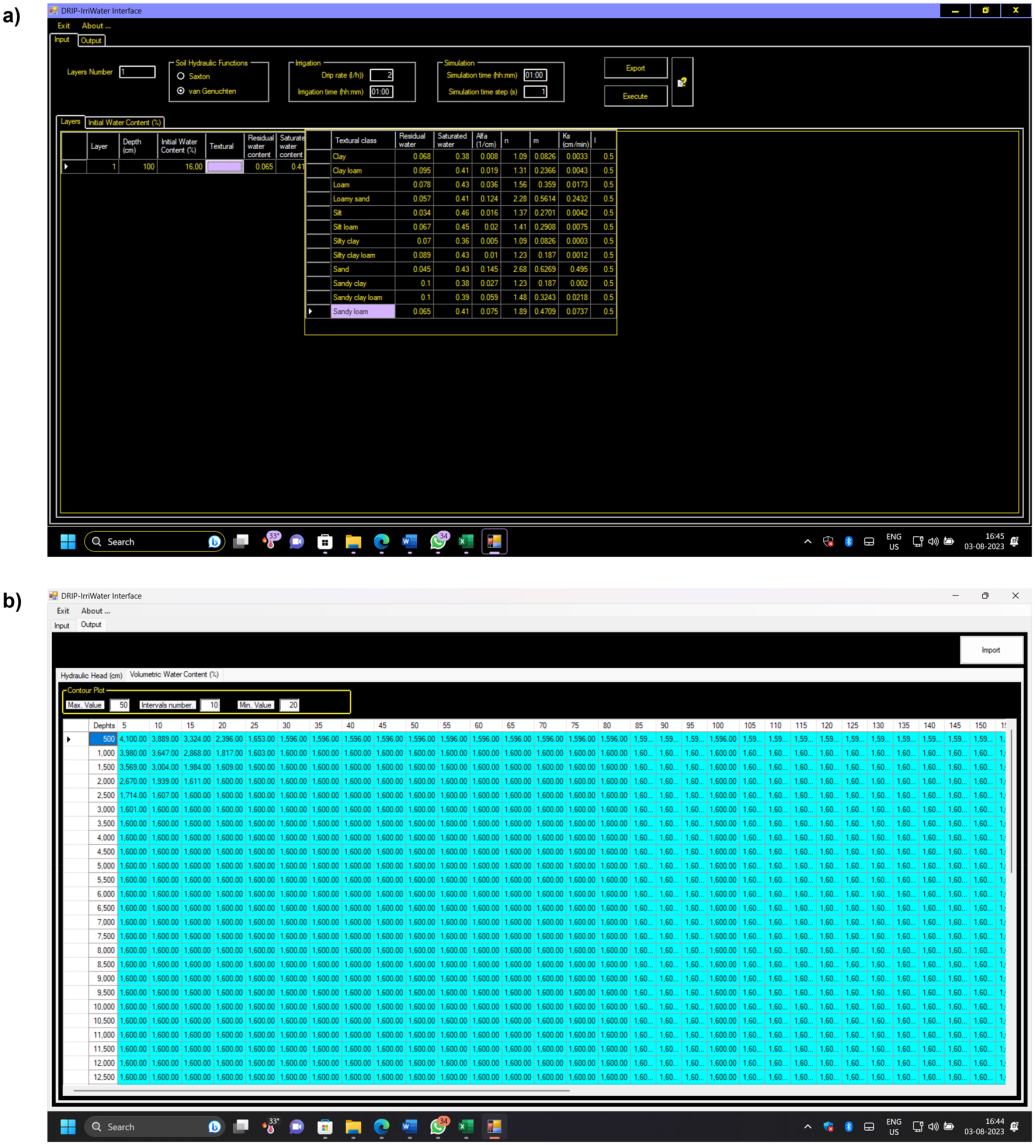
(**a**) Input and selection of the textural class and irrigation parameters following filed data and (**b**) output screen [Output] showing the values for the hydraulic potential [Hydraulic head (cm)] and volumetric water content [Volumetric water content %)].

The software program for the mathematical technique detailed in the numerical procedure section of the code and the graphical user interface (GUI) for the user's convenience make up Drip-Irriwater, which was developed by Arbat et al*.*^47^. This program's graphical user interface (GUI) was created using the Microsoft.NET framework and coded in C#. The results (distribution of soil water and hydraulic potential) are displayed in this program as soon as the input variables are entered. A FORTRAN program was made using Compaq Digital Visual Fortran 6.0 to carry out the numerical procedure and apply the numerical technique. The Input, selection of the textural class and irrigation parameters in accordance filed data and output screen illustrated in Fig. 6.

The Drip Irri-water software user interface contains three main categories of input parameters, which can be divided into Soil input parameters, Irrigation parameters, and Simulation parameters.

- The number of soil layers, the hydraulic model to be applied when calculating the hydraulic function of the soil, and the initial water content of each soil layer are examples of soil input parameters. Two alternative models, Saxton and van Genuchten–Mualem, can be used to account for soil hydraulic functions. If the Saxton model is chosen, the user must provide the sand, clay, and loam ratios in each soil layer. In contrast, the van Genuchten–Mualem model requires the selection of a textural class with the option to change some or all of the values.
- Irrigation parameters include irrigation time and emitter discharge.
- Simulation parameters include the simulation span, how many hours after irrigation the status of moisture movement and hydraulic head should be determined, and the time-step for the numerical solution, with a suggested value of 1 s.

### Criterion for statistical analysis

The performance of the Drip-Irriwater model was evaluated by comparing statistical parameters between simulated and field observed data, utilizing metrics such as mean bias error (MBE), mean absolute error (MAE), root mean square error (RMSE), mean absolute percentage error (MAPE), Pearson coefficient, R-squared correlation (R^2^), and Nash Sutcliffe model efficiency (NSE). These parameters were calculated using the following relationships^10, 72–77^.

#### Mean absolute error (MAE)

It is a common statistical metric used to assess the accuracy of a model or prediction system. It measures the average magnitude of errors between predicted values and their corresponding true or observed values. MAE is particularly useful when dealing with numerical data or regression problems.

10 $$MAE=\left(\frac{1}{N}\sum_{i=1}^{N}\left|{d}_{i}\right|\right)$$

In Eqs. (10–16), the notation N is the number of ith data sets, and d_i_ is the difference between ith predicted, and observed values; RSS = sum of squares of residuals; TSS = total sum of squares. The MAE is always a non-negative value, and its range extends from 0 to positive infinity (∞). It displays the average absolute difference between the actual values and the values that were predicted. Unlike other error metrics (e.g., Mean Squared Error or Root Mean Squared Error), the MAE is less sensitive to outliers since it only considers the absolute differences^78–80^.

#### Mean bias error (MBE)

Mean bias error is a statistical metric used to assess the accuracy and performance of a model or prediction system. It measures the average difference between the predicted values and the corresponding observed or true values. The MBE indicates whether the model tends to overestimate or underestimate the true values on average.

11 $$MBE=\left(\frac{1}{N}\sum_{i=1}^{N}{d}_{i}\right)$$

The MBE has a range from negative infinity to positive infinity. A positive MBE indicates that the model tends to overestimate the true values, while a negative MBE suggests that the model tends to underestimate the true values. Ideally, a perfect model or prediction system would have an MBE of zero, meaning it neither overestimates nor underestimates the true values on average^81, 82^.

#### Mean absolute percentage error (MAPE)

It measures the percentage difference between the predicted values and their corresponding true or observed values, providing a relative measure of the prediction error. The MAPE is always a non-negative value, and its range extends from 0% to positive infinity.

12 $$MAPE=\left(\frac{1}{N}\sum_{i=1}^{N}\left|\frac{{Forecast\, value}_{i}-{Actual \,value}_{i}}{{Actual\, value}_{i}}\right|\right)$$

A MAPE of 0% indicates that the model's predictions are perfect, meaning they exactly match the true values. However, achieving a MAPE of 0% is rare in real-world scenarios. It's important to note that MAPE has its limitations, especially when dealing with small or zero true values^83, 84^.

#### Root mean square error (RMSE)

The root mean square error (RMSE) measures the average magnitude of errors between the predicted values and their corresponding true or observed values. RMSE is particularly valuable for penalizing larger errors, making it sensitive to outliers in the data. The RMSE is give as:

13 $$RMSE=\sqrt{\left(\frac{1}{N}\sum_{i=1}^{N}{{d}_{i}}^{2}\right)}$$

The RMSE is always a non-negative value, and its range extends from 0 to positive infinity. It represents the square root of the average of the squared differences between the predicted values and the true values. By taking the square root, the RMSE is expressed in the same units as the original data, making it easier to interpret^10, 72, 85^.

#### Pearson correlation coefficient (R)

It is used to measure the strength and direction of the linear relationship between two continuous variables. It is a popular measure of correlation and is widely used in various fields, including statistics, data analysis, and machine learning. The Pearson correlation coefficient (R) is given as:

14 $$\mathrm{Pearson \,correlation\, coefficient}=\frac{\sum \left({Forecast \,value}_{i}-Mean \,forecast \,value\right)\left({Actual\, value}_{i}-Mean \,Actual \,value\right)}{\sqrt{\sum \left({Forecast \,value}_{i}-Mean\, forecast \,value\right)\sum \left({Actual\, value}_{i}-Mean\, Actual \,value\right)}}$$

The R takes values between−1, which indicates a strong linear relationship where one variable increases as the other decreases. The points in a scatter plot will align perfectly on a downward-sloping line. A R of 1 indicates a strong linear relationship where one variable increases as the other increases. If the R is greater than 0.9 and falls between 0.75 and 0.9, the correlation is considered good. Similar to this, a correlation is considered poor if its value is less than 0.6 and is considered moderate if it is between 0.6 and 0.75 ^86–88^.

#### Coefficient of determination (R^2^)

The coefficient of determination, is a statistical metric used to assess the goodness of fit of a regression model. It represents the proportion of the variance in the dependent variable (the one being predicted) that is explained by the independent variable(s) used in the model. The coefficient of determination (R^2^) is given as:

15 $${R}^{2}=1-\frac{RSS}{TSS}$$

R^2^ has a range from 0 to 1. The values of R^2^ can be interpreted as if R^2^ = 1 refer as “Very good”, 0.8 ≤ R^2^ < 1 as “Good”, 0.6 ≤ R^2^ < 0.8 as “Satisfactory”, R^2^ < 0.6 as “Weak”, R^2^ = 0 as “Inefficient” and R^2^ < 0 as “Invalid”^89, 90^.

#### Nash Sutcliffe model efficiency (NSE)

It is a frequently employed statistical metric for assessing how well hydrological or hydrodynamic models perform. It assesses the ability of a model to predict observed values and is commonly applied in hydrology, hydrogeology, and water resources engineering. The Nash–Sutcliffe model efficiency (NSE) is given as:

16 $$NSE=1-\frac{\sum_{i}^{N}{\left({Forecast \,value}_{i}-{Actual \,value}_{i}\right)}^{2}}{\sum_{i}^{N}{\left({Actual \,value}_{i}-\overline{Mean \,Actual \,value }\right)}^{2}}$$

Values between 0.75 and 1.0 for the NSE are considered "very good," Regarding the performance of the model, values between 0.4 and 0.5 may be described as "acceptable," 0.65 to 0.75 as "good," and 0.65 to 0.65 as "satisfactory^73, 90–92^.

## Result

### Comparison of soil moisture distribution

**Figure 7:**
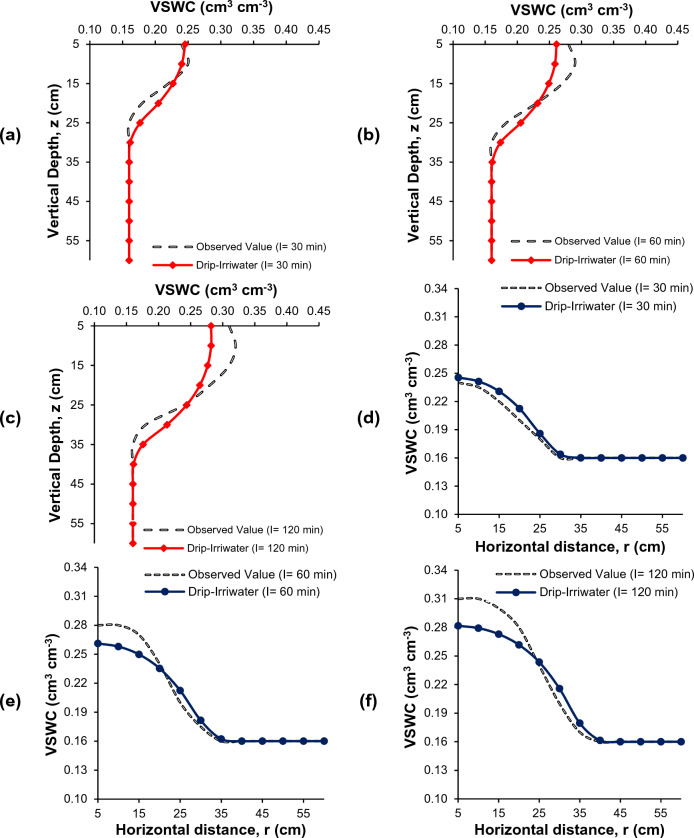
Comparison of volumetric soil water content (VSWC) Observed (black dot) and Drip-Irriwater (red line & blue line): vertically (**a–c**) and horizontally (**d–f**) corresponding to the emitter flow rate of 2.0 L h^−1^ for an irrigation duration of 30, 60 and 120 min, of 1 h later stopping irrigation respectively.

**Figure 8:**
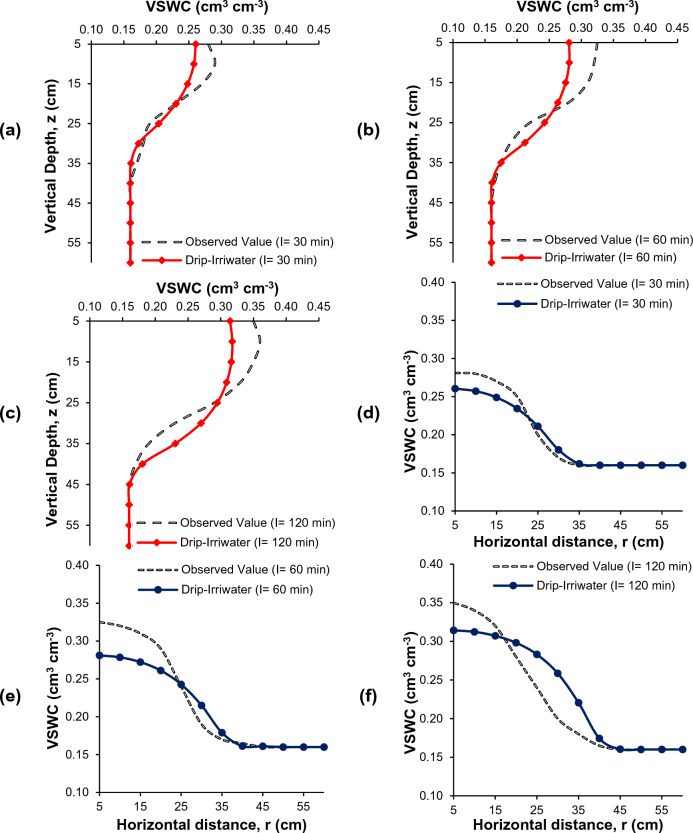
Comparison of volumetric soil water content (VSWC) Observed (black dot) and Drip-Irriwater (red line & blue line): vertically (**a–c**) and horizontally (**d–f**) corresponding to the emitter flow rate of 4.0 L h^−1^ for an irrigation duration of 30, 60 and 120 min, of 1 h later stopping irrigation respectively.

**Figure 9:**
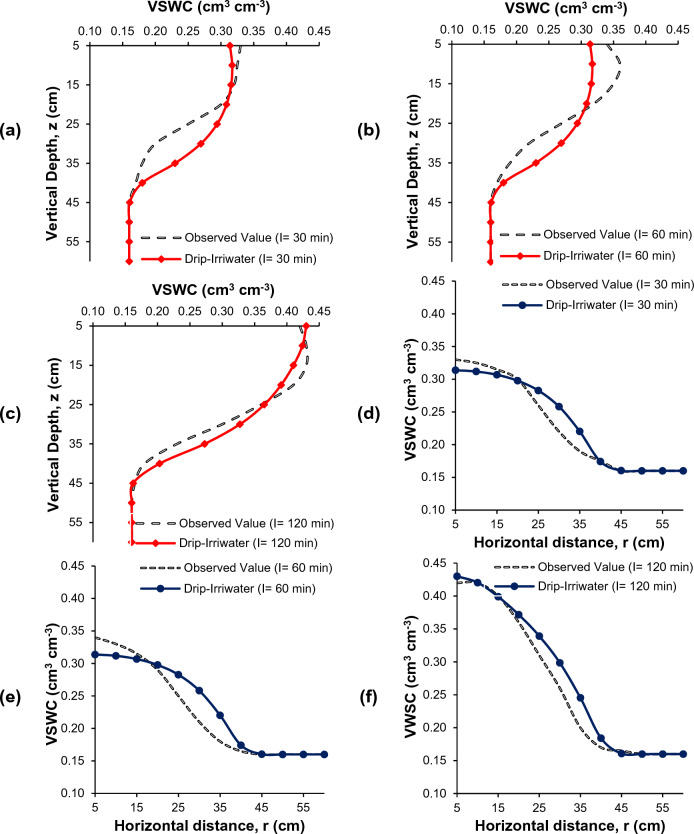
Comparison of volumetric soil water content (VSWC) Observed (black dot) and Drip-Irriwater (red line & blue line): vertically (**a–c**) and horizontally (**d–f**) corresponding to the emitter flow rate of 8.0 L h^−1^ for an irrigation duration of 30, 60 and 120 min, of 1 h later stopping irrigation respectively.

Figures 7, 8 and 9 present the comparison between measured and simulated (using Drip-Irriwater) volumetric water content distributions in a field study of drip irrigation. The irrigation time was 30, 60, and 120 min, with emitter discharge rates of 2, 4, and 8 L h^−1^. At the start of irrigation, the volumetric soil moisture content was around 0.16 cm^3^ cm^−3^, close to the permanent wilting point. Each figure displays the measured and simulated volumetric water content for the different treatment combinations in a chosen soil profile. The simulated water content distributions closely match the observed, as demonstrated by the transect plot results.

Each figures display the actual and expected water content for a specific profile. When comparing the predicted soil moisture distribution with the observed values in the soil's wetted zone (60 cm vertically and 60 cm horizontally from the emitter point), the figure shows a comparison of the volumetric soil water content vertically and laterally at 5 to 60 cm depth from the topsoil surface. This corresponds to a drip flow of 2.0 L h^−1^ for irrigation times of 30, 60, and 120 min, respectively, 1 h after the termination of irrigation (Fig. 7). Similarly, Figs. 8 and 9 show the drip flow rates of 4 and 8 L h^−1^, respectively.

It is clear from Figs. 7, 8 and 9 that the volumetric soil moisture content near the dripper emission point was higher after one hour of irrigation in the experimental plots and decreased as the distance from the dripper increased. The moisture content also increased to a certain depth in the vertical direction before decreasing. For example, after 1 h of irrigation using 2 L h^−1^ for 30 min, the volumetric soil water content near the dripper point (at 5 cm depth) was 0.24 cm^3^ cm^−3^ and increased to 0.25 cm^3^ cm^−3^ at a depth of 10 cm but then decreased to 0.22, 0.18, and 0.16 cm^3^ cm^−3^ at 15, 20, and 25 cm depth, respectively. Similarly, in the horizontal direction, near the dripper point (at 5 cm depth) was 0.24 cm^3^ cm^−3^ and decreased to 0.23, 0.22, 0.20, 0.18, and 0.16 at 10, 15, 20, 25, and 30 cm, respectively. A similar trend in moisture content was observed for 60 and 120 min of irrigation duration, one hour after irrigation. However, the moisture level was increasing in the wetted zone.

Similar trends were found in 4 and 8 L h^−1^ emitter discharge rates for 30, 60, and 120-min irrigation. However, the only difference is changes in volumetric soil water content. The wetted soil width and depth were identical in loam soil when the same amount of water was applied. However, the spatial distributions of the volumetric water content were not significantly different, i.e., moisture content was almost similar.

The accuracy of Drip-Irriwater and the obtained results are summarized in Table 4. The volumetric water contents were forecasted using the Drip-Irriwater program code. Those measured at the irrigation stopped are compared in Table 4 and indicate that the average MAE was 0.0181 cm^3^ (lowest value 0.011 and highest 0.022), MBE was 0.0005 cm^3^ (lowest value −0.0008 and highest 0.011), MAPE was 7.3 (lowest value 4.6 and highest 8.8), RMSE was 0.023 cm^3^ cm^−3^ (lowest value 0.015 and highest 0.028), Pearson coefficient was 0.95 (lowest value 0.92 and highest 0.977), a mean R^2^ of 0.91 (lowest value 0.84 and highest 0.95), and NSE was 0.887 (lowest value 0.828 and highest 0.921). The values of NSE for Drip-Irriwater models were higher than 0.75 showing high precision in predicting soil moisture movement in both directions. R^2^ values were found more 0.8 in all cases and proved that the model has high degree of goodness of fit. Whereas the Pearson correlation coefficient (R) were greater than 0.9 and found strong correlation between observed and predicted value. The MAE, MBE, MAPE, and RMSE values was very low and stating excellent accuracy in predicting soil moisture movement.

**Table 4:** Performance of MAE, MBE, MAPE, RMSE, Pearson coefficient, and R^2^ NSE statistics between the measured and forecasted soil water contents with an applied irrigation volume 24 h after the irrigation stopped.

Flow rate (L h^−1^)	MAE	MBE	MAPE (%)	RMSE (cm^3^ cm^−3^)	Pearson coefficient	R^2^	NSE
Wetted soil width							
2	0.011	−0.003	4.60	0.015	0.977	0.9551	0.919
4	0.022	−0.003	8.70	0.027	0.921	0.8490	0.828
8	0.018	0.011	7.40	0.023	0.921	0.9397	0.921
Wetted soil depth							
2	0.016	−0.002	6.80	0.020	0.977	0.9377	0.882
4	0.020	−0.008	7.50	0.025	0.954	0.9098	0.870
8	0.022	0.008	8.80	0.028	0.958	0.9183	0.904
Average	0.0181	0.0005	7.3	0.023	0.951	0.9182	0.887

*MAE* mean absolute error, *MBE* mean bias error, *MAPE* mean absolute percentage error, *RMSE* root mean square error, *NSE* Nash Sutcliffe model Efficiency coefficient, *r*^*2*^ coefficient of correlation.

**Figure 10:**
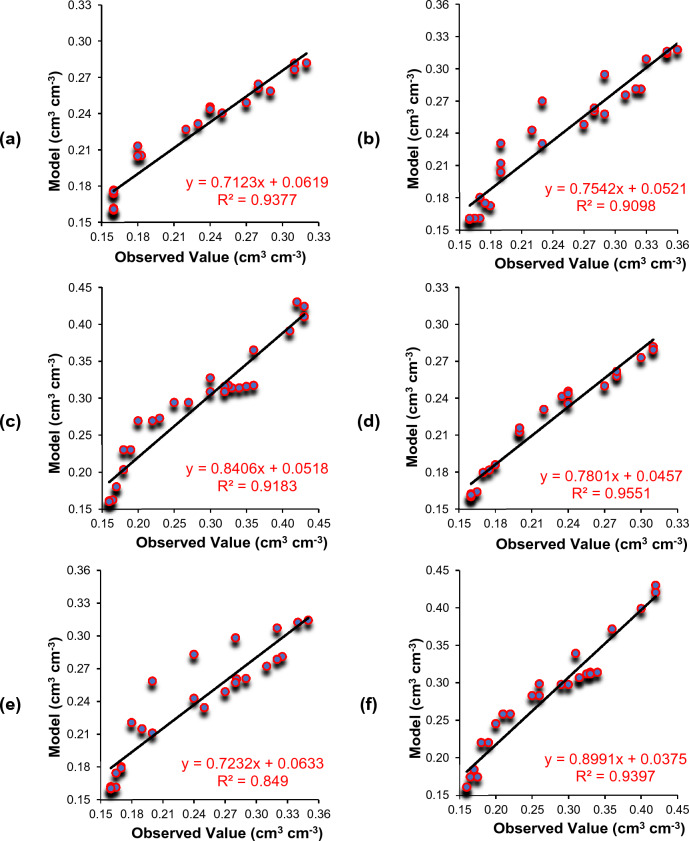
Statistics comparison between measured VSWC with estimated (Drip-Irriwater) for (**a–c**) wetted soil depth using 2, 4 and 8 L h^−1^ emitter flow rate & (**d–f**) wetted soil width using 2, 4 and 8 L h^−1^ emitter discharge rate.

The drip-Irriwater program code estimates volumetric water content in descending order from top to bottom. However, the measured volumetric water content was highest in the soil layer's top 10–15 cm (Figs. 7, 8 and 9). This could be caused by *evaporation* from the upper layer into the atmosphere or *Infiltration* from the top layer into the lower layer^9, 47, 93^. In the validation stage for estimation of volumetric soil water contents, scatter plots between the measured and forecasted with Drip-Irriwater with varying emitter discharge rate and irrigation duration are presented in Fig. 10. With an average mean coefficient of determination (R^2^) of 0.91, Fig. 10 shows excellent agreement between measured and forecasted volumetric soil water contents. Our results follow Arbat et al*.*^47^, showing good agreement between simulated and field observations. Consequently, using Drip-Irriwater allows the simulation of water content distribution in soil using vertical or horizontal moisture irrigation.

### Comparison of wetted soil width and depth in field observation

**Figure 11:**
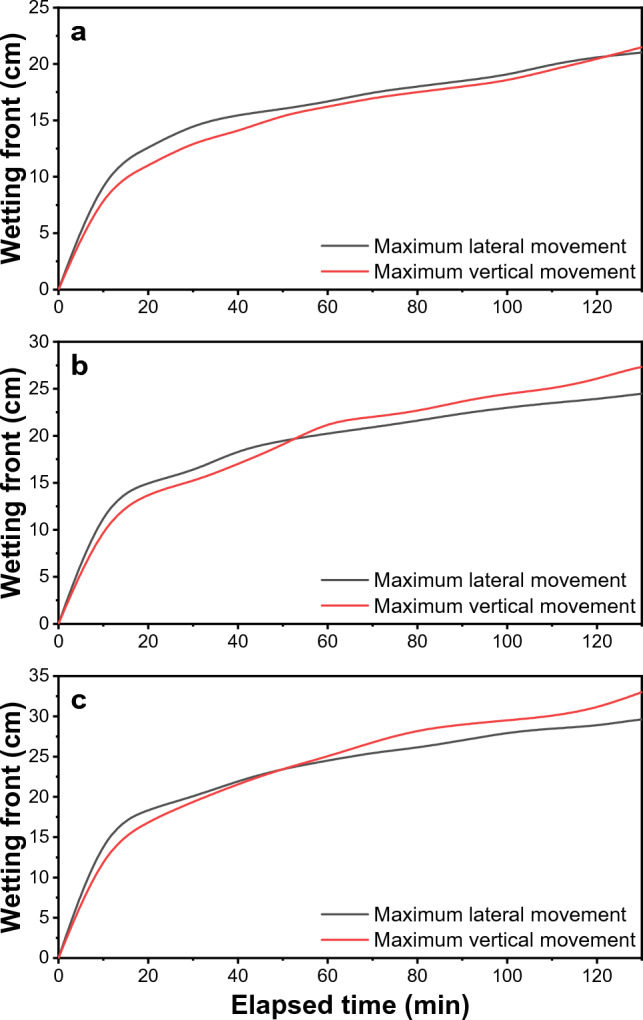
Measured wetting pattern dimensions in average loam soils as a function of the volume of applied water (L) for different rates: (**a**) 2, (**b**) 4, and (**c**) 8 L h^−1^. After the irrigation was stopped, the wetting pattern's final dimensions were measured.

The following wetting curves were generated based on the outcomes of the field experiments conducted as shown in Fig. 11. Table 5 shows a statistical comparison of wetted soil width and depth determined at the end of irrigation using 2, 4, and 8 L h^−1^ emitter discharge rates during different irrigation time durations. In the 2 L h^−1^ emitter discharge rate at 30 min of the irrigation, the front edge of soil moisture vertically reached a depth of 13.10 cm. It laterally distributed to 14.65 cm around the emitter. At 60 and 120 min, the vertical distribution of soil moisture was about 16.20 cm and 20.44 cm, while the horizontal distribution was about 16.65 and 20.62 cm, respectively. For 4 and 8 L h^−1^ discharge rates at 30, 60, and 120 min of irrigation, the front edge of soil moisture vertically reached the depth of 15.10, 21.50, and 26.00 cm; 19.40, 25.00, and 31.00, respectively, and laterally distributed to 16.20, 20.25 and 23.90; 20.00, 24.50 and 28.81 cm respectively around the emitter. The influence of several types of emitters on observed wetted soil radius and depth was considerable, as shown in Table 5. From Table 5, it is evident that a significant difference was found in emitter discharge rate (E) and irrigation duration (I) in the front edge of soil moisture was vertical and horizontal. However, in the interaction ((E × I) between emitter discharge rate (E) and irrigation duration (I), no significant difference was found at the critical difference at 0.05 percent level of significance, which means that, if we apply the same volume of irrigation, i.e., 4 L in each case (2 L h^−1^ for 120-min, 4 L h^−1^ for 60-min and 8 L h^−1^ for 30-min duration) front edge of soil moisture in vertically and well as horizontally will not be significant.

**Table 5:** Statistics of comparison between measured and forecasted wetted soil radii and depth with an irrigation volume after the irrigation event stopped.

Soil type	Time duration of irrigation	Flow rate (L h^−1^)	Measured width (cm)	Forecasted width (cm)	Measured depth (cm)	Forecasted depth (cm)	C.D. for measured value of wetted soil width	C.D. for measured value of wetted soil depth
Loam	30 min	2	14.65	20.00	13.10	20.00	Emitter discharge rate (E): 1.3728 Time duration of irrigation (I): 1.3728 Interaction (E × I): N.S	Emitter discharge rate (E): 2.2213 Time duration of irrigation (I): 2.2213 Interaction (E × I): N.S
	60 min	2	16.65	20.00	16.20	20.00		
	120 min	2	20.62	25.00	20.44	25.00		
	30 min	4	16.20	20.00	15.10	20.00		
	60 min	4	20.25	25.00	21.50	20.00		
	120 min	4	23.90	25.00	26.00	25.00		
	30 min	8	20.00	20.00	19.40	20.00		
	60 min	8	24.50	25.00	25.00	20.00		
	120 min	8	28.81	25.00	31.00	25.00		

C.D. is the critical difference at a 0.05 percent level of significance.

*N.S.*  not Significant.

24 After the end of irrigation, there was a huge vertical and horizontal water movement. However, the horizontal movement was smaller than the vertical movement due to the capillary effect and forces. The maximum wetting depth of 23.00, 35.00, and 40.00; 27.00, 40.00, and 45.00; 32.00, 40.00, and 45.00 was observed at 30, 60, and 120-min irrigation duration using 2, 4, and 8 L h^−1^ emitter flow rate respectively 24 after the irrigation event stooped (Table 6). Table 6 shows a statistical comparison of wetted soil radius and depth recorded 24 h after irrigation completion using 2, 4, and 8 L h^−1^ emitter discharge rates and different irrigation time durations. The drip-Irriwater program code predicts a slightly larger wetted soil width than the steel tape-based (observed) wetted width, with a mean absolute deviation of 3.00 cm at the irrigation closing and 5.0 cm after 24 h of allocation (Tables 5 and 6). The Drip-Irriwater program code's calculation of the wetted soil depth was slightly greater than that determined by steel tape measurements during irrigation durations of 60 and 120 min, with a mean absolute difference of 3.8 cm after irrigation and 6.33 cm after 24 h of moisture redistribution. After 24 h of irrigation, there were fewer differences between the predicted wetted soil width and depth and those determined using steel tape. Table 5 demonstrates that for loam soil in field experiments, the wetted soil depth was marginally more than the wetted soil breadth at the beginning of irrigation but much less so as irrigation time duration rose. The predicted wetted soil width and depth for loam soil were equal (measured with the Drip-Irriwater code program, v1.0).

**Table 6:** Comparison between the measured and forecasted wetted soil width and depth with an applied irrigation volume 24 h after the irrigation event stopped.

Time duration of irrigation	Discharge rate (L h^−1^)	Measured width (cm)	Forecasted width (cm)	Measured depth (cm)	Forecasted depth (cm)
30 min	2	25.00	35.00	23.00	35.00
	4	30.00	35.00	35.00	35.00
	8	40.00	45.00	40.00	45.00
60 min	2	30.00	35.00	27.00	35.00
	4	40.00	45.00	40.00	45.00
	8	40.00	45.00	45.00	45.00
120 min	2	35.00	40.00	32.00	40.00
	4	40.00	45.00	40.00	45.00
	8	45.00	45.00	45.00	45.00

### Horizontal and vertical wetting front and soil moisture distribution profile under different treatment just at the end of irrigation and 24 h after starting irrigation

**Figure 12:**
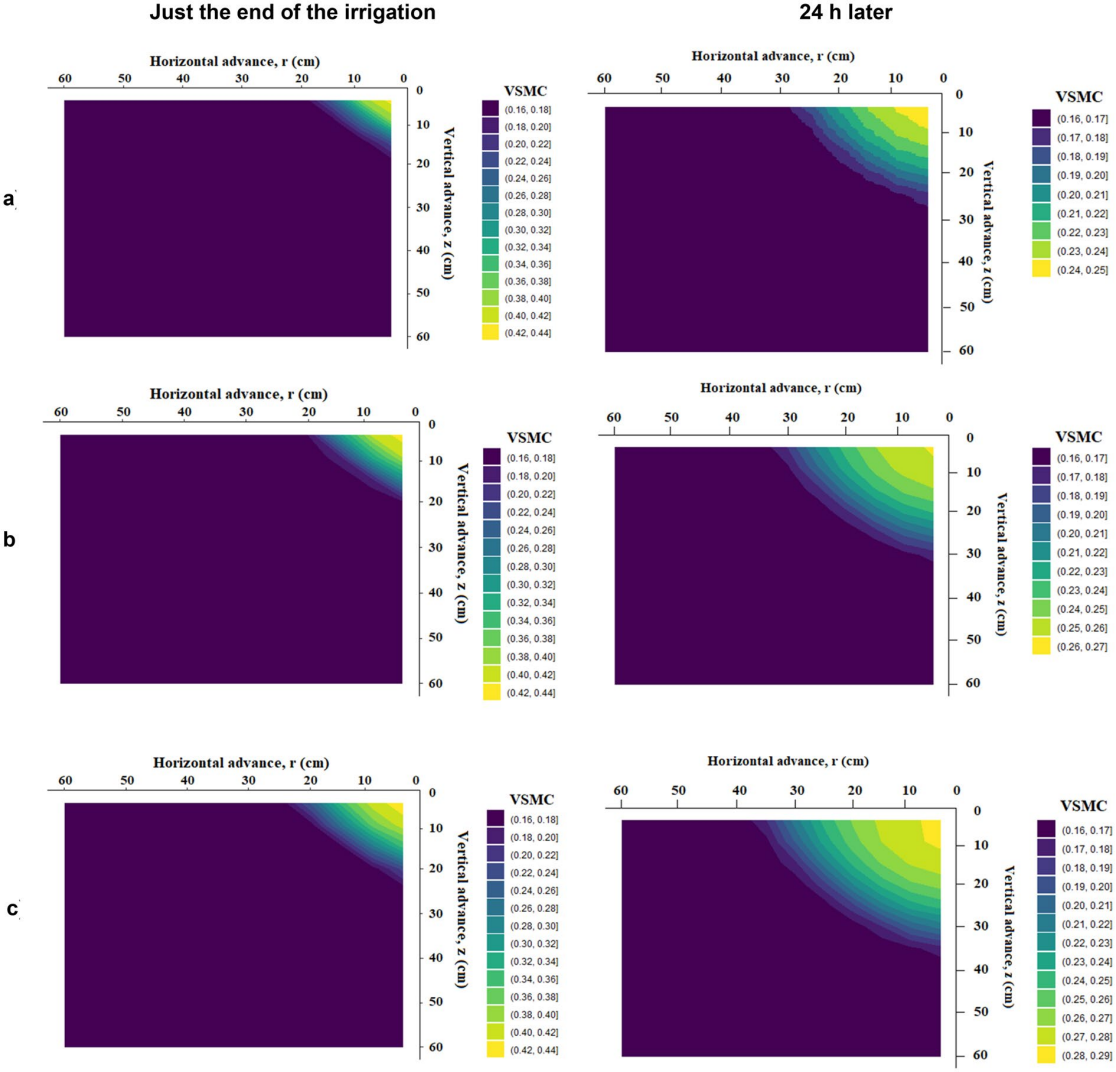
Forecasted VSWC dynamics at just the end of irrigation and 24 h later with an emitter flow rate of 2 L h^−1^ and irrigation period of (**a**) 30 min, (**b**) 60 min, and (**c**) 120 min, respectively.

**Figure 13:**
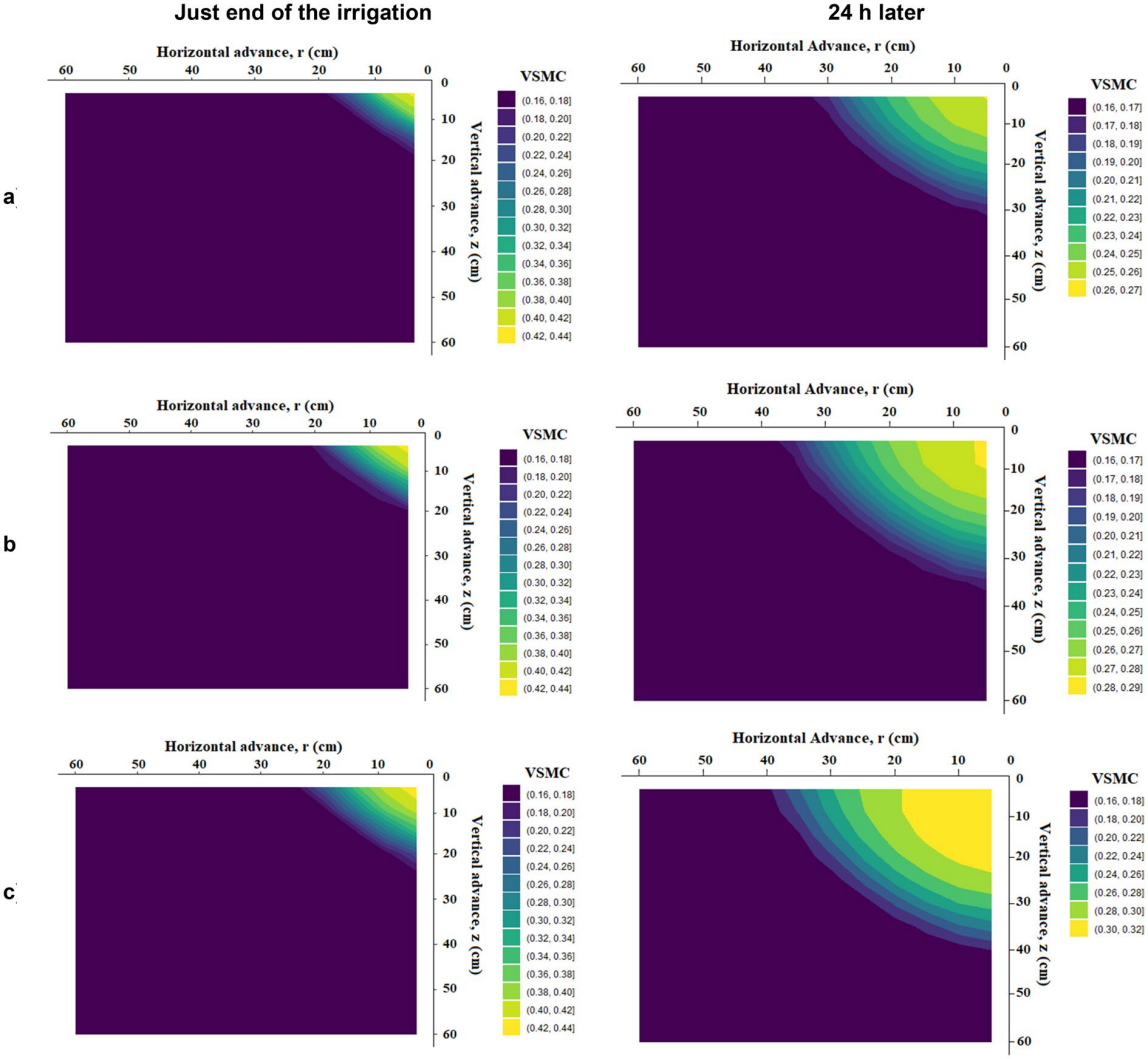
Forecasted VSWC dynamics at just the end of irrigation and 24 h later with an emitter flow rate of 4 L h^−1^ and irrigation period of (**a**) 30 min, (**b**) 60 min, and (**c**) 120 min, respectively.

**Figure 14:**
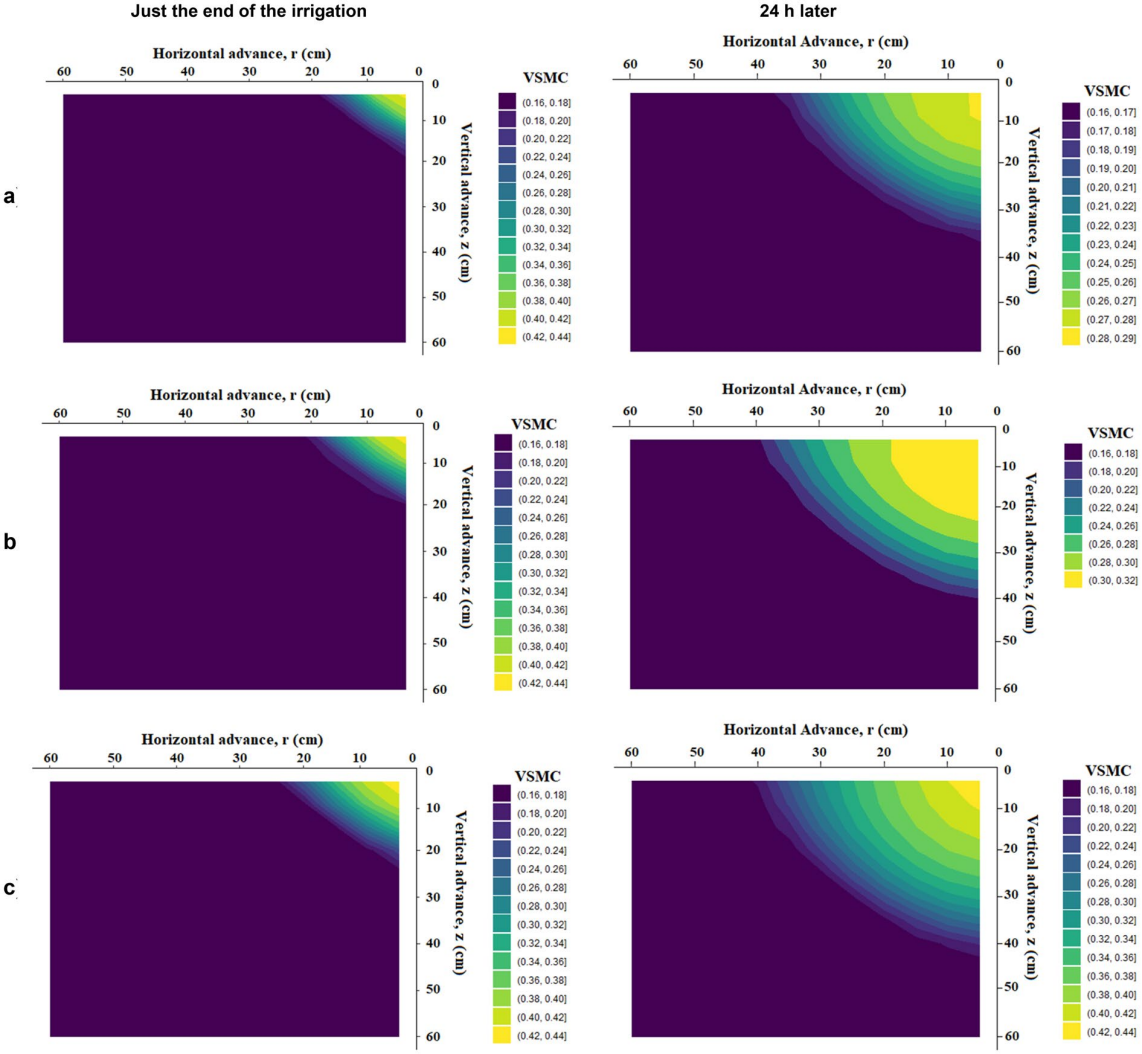
Forecasted VSWC dynamics at just the end of irrigation and 24 h later with an emitter flow rate of 8 L h^−1^ and irrigation period of (**a**) 30 min, (**b**) 60 min, and (**c**) 120 min, respectively.

The distribution or movement directions of soil moisture were useful information in water management practices. Figures 12, 13 and 14 show volumetric soil moisture profiles in loam soil with an initial volumetric soil moisture content of 0.16 cm^3^ cm^-3^ for emitter discharge rates of 2, 4, and 8 l h^–1^ and irrigation times of 30, 60, and 120 min, with one just at the end of irrigation and the other 24 h later. The Drip-Irriwater program code determines the temporal distribution patterns of soil water content at three distinct times (30, 60, and 120 min). In all cases investigated, horizontal and vertical wetting front at the end of irrigation is lower than 24 h after starting irrigation. Two forces influence water distribution, capillary action exerted by the micro-pores in the soil and gravity force. Due to these effects, a significant difference was found just after the end of irrigation and 24 after the end of irrigation. Figure 12 shows that the horizontal and vertical wetting fronts are lower at the end of irrigation than they were 24 h after starting irrigation in both cases for loam soil. i.e., due to the same volume of water flowing from the emitter. There is a clear difference in the moisture content of the wetted bulb at horizontal and vertical intervals immediately after irrigation and after 24 h. This can be explained by Table 5, which shows that the relationship between emitter discharge rate and irrigation duration for both width and depth was insignificant for equal flow volume. Because of the same volume of water flow from the emitter, similar phenomena were observed in Figs. 13 and 14. The initial soil moisture content, hydraulic features of the soil, emitter discharge rate, emitter lines spacing, root water uptake spatial, irrigation depth, and temporal distribution all have a significant impact on soil moisture content spatial distribution under point source drip irrigation. In sandy clay loam soil with more micropores (52% porosity), water was likely to move more horizontally due to capillary action than vertically.

In contrast, loamy sand soil, dominated by macrospores water, tended to move more vertically due to gravity. The soil was wetted to a greater depth as the same volume of water was applied. Every irrigation event aims to apply water to the root zone at the ideal moisture content. The irrigation time must be adjusted according to the depth of active roots and the rate of water evaporation. The intergranular holes are smaller, the permeability is less, and the capillary action is stronger the finer the soil texture. This has the effect of making the vertical and horizontal motion of the wetting front more uniform.

## Discussion

### Comparison of soil moisture distribution

Optimizing the flow rate of the emitter could lead to an increase in water use efficiency under apple cultivation^94, 95^. A lower water flow rate is not enough for plant growth, while with a very high flow rate, the water reaches the end faster and less water penetrates the soil layers and is lost through deep infiltration^96^. Hence the wetting front and soil moisture distribution in soil must be match with characteristics of plant root distribution to enhance the water use efficiency and save water. Wetted width and depth and soil mositure were affected by the discharge rate of the emitter. With the increasing flow rate of water, the depth and breadth of the wetted soil increased. The reason for this was that with increasing discharge rates, the amount of water supplied in a given period increases, creating a higher wet ground area. Wetted width and depth were observed to be affected by the duration of the water supply. It was increased with water usage time for a given discharge rate. This was because the longer the duration of operation, the more water was applied and the large amount of soil has absorbed that water. The same trend of soil moisture under different flows was also observed by Al-Ogaidi et al.^35^ and Jamei et al.^12^.

### Comparison of wetted soil width and depth in field observation

When applying 8 L h^-1^ of water, the volumes and discharge rate were found to be sufficient to meet the maximum evapotranspiration demand and allow for greater vertical and horizontal extension of the wetting area. Significant changes in water content at different soil depths with changes in flow rate and duration were associated with proper distribution and longer water opportunity times. This conclusion is similar to that of Sun et al.^14^ and Li et al.^97^. Soil vertical wetting circle expanded to more than 20 cm in 120 min of water flow. This also proved that in drip irrigation, soil water mainly moves from the 20–30 cm deeper soil layer. Therefore, it could be concluded that subsurface drip irrigation allows for more effective use of the water in the root zone for plant growth. This might be as a result of the system's low evaporative loss.

### Horizontal and vertical wetting front and soil moisture distribution profiles under different treatment

When designing a drip irrigation system, consideration must be given to the width and depth of the wet soil zone. The physical characteristics of the soil, drip irrigation parameters like drip emitters, the flow rate, and the amount of water used were primarily related to the geometry of the wet soil's dimensions^9, 10^. The depth and width of the soil in the wetted zone increased as the water supply increased. Similar patterns in the effects were also seen across all placement depths. The water moves more downward as the wetting front approaches the bottom surface. The increased evaporation at the soil's surface may have contributed to the lower wetting of the upper layers, and the gravitational movement of water beneath the sandy loam soil was more capillary, with water moving from the upper to lower layers. The maximum moisture content was close to the emitting point and decreased as the distance from the emitter increased, in addition to the vertical waterfront advance. Maurice et al.^98^ reported a finding of a similar nature.

### Significant shortcomings and future research scope

This research has been carried out in only one type of soil (loamy soil), to see the effect of different discharge rate and irrigation time, research should be done in different types of soil. Instead of laboratory or field work, measurement and analysis of soil moisture movement and wetting front in crop can be done to see effect of crop/plant root zone on the soil moisture movement and wetting front, which can be interpreted in real situations. It will be more beneficial to choose the application time according to the crop water requirement instead of randomly setting the irrigation time. Keeping all these things in mind, there are limitless possibilities for future research, and a good conclusion can be made. Along with moisture movement, crop/plant yield and plant parameters can also be compared in future research.

## Conclusions

In the present study, the Drip-Irriwater code was used to estimate soil wetting dimension (i.e., depth and width of wetted soil) and soil moisture redistribution under surface trickle irrigation systems for effective and efficient irrigation system design. Three different capacity emitters (2, 4, and 8 l h^−1^) with varying time durations were used to evaluate horizontal and vertical advancement of wetted soil from a single point source of surface drip irrigation (30, 60, and 120 min) just after application and 24 h later of irrigation. The Drip-Irriwater (v1.0) model simulated soil moisture distribution with observed data. According to statistical analysis results (MAE, MBE, MAPE, RMSE, NSE, and R^2^), the estimated value of moisture dynamics (soil moisture redistribution and vertical and horizontal wetting front advance surface drip irrigation) was found quite close to the observed value. In all circumstances, the proportion of moisture dispersal increased as the emitter flow rate from the emitting site increased (before and after 24 h of irrigation). An accurate assessment of waterfront movement can improve water usage efficiency (WUE) and economic factors in cropping systems by applying a precise amount of water to crop root zones. As a result, because the input parameters are gently known or calculated, Drip-Irriwater computer software (v1.0) could be hugely beneficial and is available to drip system design engineers, irrigation specialists, and operators in the public domain.

## Data Availability

The datasets used and/or analyzed during the current study are available from the corresponding author on reasonable request.

## References

[1] Jha SK (2017). Root development and water uptake in winter wheat under different irrigation methods and scheduling for North China. Agric. Water Manag..

[2] Jat R (2022). Deficit irrigation scheduling with mulching and yield prediction of guava (*Psidium guajava* L.) in a subtropical humid region. Front. Environ. Sci..

[3] Kumar M, Kumar R, Rajput TBS, Patel N (2017). Efficient design of drip irrigation system using water and fertilizer application uniformity at different operating pressures in a semi-arid region of India. Irrig. Drain..

[4] Kumar R, Kumar M (2020). Effect of drip irrigated mulch on soil properties and water use efficiency—A review. J. Soil Water Conserv..

[5] Kumar R, Shankar V, Jat MK (2015). Evaluation of root water uptake models—A review. ISH J. Hydraul. Eng..

[6] Ma X, Sanguinet KA, Jacoby PW (2020). Direct root-zone irrigation outperforms surface drip irrigation for grape yield and crop water use efficiency while restricting root growth. Agric. Water Manag..

[7] Wang Y (2020). Effect of a root-zone injection irrigation method on water productivity and apple production in a semi-arid region in north-western China. Irrig. Drain..

[8] Subbaiah R (2013). A review of models for predicting soil water dynamics during trickle irrigation. Irrig. Sci..

[9] Vishwakarma DK (2022). Evaluation and development of empirical models for wetted soil fronts under drip irrigation in high-density apple crop from a point source. Irrig. Sci..

[10] Vishwakarma DK, Kumar R, Tomar AS, Kuriqi A (2023). Eco-hydrological modeling of soil wetting pattern dimensions under drip irrigation systems. Heliyon.

[11] Elbeltagi, A., Kushwaha, N. L., Srivastava, A. & Zoof, A. T. Artificial intelligent-based water and soil management. in *Deep Learning for Sustainable Agriculture* (eds. Poonia, R. C., Singh, V. & Nayak, S. R. B. T.-D. L. for S. A.). 129–142. 10.1016/B978-0-323-85214-2.00008-2 (Elsevier, 2022).

[12] Jamei M (2023). A comprehensive investigation of wetting distribution pattern on sloping lands under drip irrigation: A new gradient boosting multi-filtering-based deep learning approach. J. Hydrol..

[13] Rocha, M. O., dos Teixeira, A. S., das Silva Filho, F. C., da Gondim, R. S. & de Sousa, A. B. O. The use of numerical modelling to assess soil water dynamics in subsurface irrigation. *Rev. Ciênc. Agron.* **54**, 156 (2023).

[14] Sun L (2023). Simulation of soil water movement and root uptake under mulched drip irrigation of greenhouse tomatoes. Water.

[15] Fan Y, Yin W, Yang Z, Wang Y, Ma L (2023). Moisture content distribution model for the soil wetting body under moistube irrigation. Water SA.

[16] Kang S, Hu X, Jerie P, Zhang J (2003). The effects of partial rootzone drying on root, trunk sap flow and water balance in an irrigated pear (*Pyrus communis* L.) orchard. J. Hydrol..

[17] Zhang Z, Zhang Y, Shi Y, Yu Z (2020). Optimized split nitrogen fertilizer increase photosynthesis, grain yield, nitrogen use efficiency and water use efficiency under water-saving irrigation. Sci. Rep..

[18] Mahajan G, Singh KG (2006). Response of greenhouse tomato to irrigation and fertigation. Agric. Water Manag..

[19] Kumar M, Kumar R (2022). Yield response and validation of CROPWAT for baby corn under drip irrigation. J. Soil Water Conserv..

[20] Kumar R, Haroon S (2021). Water requirement and fertigation in high density planting of apples. Indian J. Hortic..

[21] Kumar M, Rajput TBS, Kumar R, Patel N (2016). Water and nitrate dynamics in baby corn (*Zea mays* L.) under different fertigation frequencies and operating pressures in semi-arid region of India. Agric. Water Manag..

[22] Zhang, F. *et al.**Integrated Nutrient Management for Food Security and Environmental Quality in China*. 1–40. 10.1016/B978-0-12-394277-7.00001-4 (2012).

[23] Evans RG, Sadler EJ (2008). Methods and technologies to improve efficiency of water use. Water Resour. Res..

[24] Cantuarias T, Tomer E (1995). Improving avocado tree water status under severe climatic conditions by increasing wetted soil volume. Proc. World Avocado Congress III.

[25] Dasberg, S. & Or, D. Practical applications of drip irrigation. in *Drip Irrigation*. 125–138. 10.1007/978-3-662-03963-2_6 (Springer, 1999).

[26] Bajpai A, Kaushal A (2020). Soil moisture distribution under trickle irrigation: A review. Water Supply.

[27] Patel N, Rajput TBS (2008). Dynamics and modeling of soil water under subsurface drip irrigated onion. Agric. Water Manag..

[28] Malek K, Peters RT (2011). Wetting pattern models for drip irrigation: New empirical model. J. Irrig. Drain. Eng..

[29] Phogat V, Skewes MA, Mahadevan M, Cox JW (2013). Evaluation of soil plant system response to pulsed drip irrigation of an almond tree under sustained stress conditions. Agric. Water Manag..

[30] Parvizi H, Sepaskhah AR, Ahmadi SH (2014). Effect of drip irrigation and fertilizer regimes on fruit yields and water productivity of a pomegranate (*Punica granatum* (L.) cv. Rabab) orchard. Agric. Water Manag..

[31] Thorburn PJ, Cook FJ, Bristow KL (2003). Soil-dependent wetting from trickle emitters: Implications for system design and management. Irrig. Sci..

[32] Yao WW, Ma XY, Li J, Parkes M (2011). Simulation of point source wetting pattern of subsurface drip irrigation. Irrig. Sci..

[33] Naglič B, Kechavarzi C, Coulon F, Pintar M (2014). Numerical investigation of the influence of texture, surface drip emitter discharge rate and initial soil moisture condition on wetting pattern size. Irrig. Sci..

[34] Bostjan NAGLIC. Numerical and experimental evaluation of wetted soil volume in surface drip irrigation systems. *Thesis* **I**, 214 (2014).

[35] Al-Ogaidi AAM, Wayayok A, Rowshon MK, Abdullah AF (2016). Wetting patterns estimation under drip irrigation systems using an enhanced empirical model. Agric. Water Manag..

[36] Al-Ogaidi AAM, Wayayok A, Kamal R, Abdullah AF (2016). Modelling soil wetting patterns under drip irrigation using Hydrus-3D and comparison with empirical models. Glob. J. Eng. Technol. Rev..

[37] Al-Ogaidi AAM, Wayayok A, Kamal MR, Abdullah AF (2015). A modified empirical model for estimating the wetted zone dimensions under drip irrigation. J. Teknol..

[38] Tikhamarine Y, Malik A, Souag-Gamane D, Kisi O (2020). Artificial intelligence models versus empirical equations for modeling monthly reference evapotranspiration. Environ. Sci. Pollut. Res..

[39] Ben-Asher J, Yano T, Shainberg I (2003). Dripper discharge rates and the hydraulic properties of the soil. Irrig. Drain. Syst..

[40] Cote CM, Bristow KL, Charlesworth PB, Cook FJ, Thorburn PJ (2003). Analysis of soil wetting and solute transport in subsurface trickle irrigation. Irrig. Sci..

[41] Cook FJ, Fitch P, Thorburn PJ, Charlesworth PB, Bristow KL (2006). Modelling trickle irrigation: Comparison of analytical and numerical models for estimation of wetting front position with time. Environ. Model. Softw..

[42] John, F. *et al.**WetUp : A Software Tool to Estimate Wetting Patterns from Drip Emitters for Better Irrigation WetUp—A Software Tool to Display Approximate Wetting*. (2006).

[43] Kandelous MM, Šimůnek J (2010). Comparison of numerical, analytical, and empirical models to estimate wetting patterns for surface and subsurface drip irrigation. Irrig. Sci..

[44] Kandelous MM, Šimůnek J (2010). Numerical simulations of water movement in a subsurface drip irrigation system under field and laboratory conditions using HYDRUS-2D. Agric. Water Manag..

[45] Subbaiah R, Mashru HH (2013). Modeling for predicting soil wetting radius under point source surface trickle irrigation. Agric. Eng. Int. CIGR J..

[46] Elmaloglou S, Soulis KX, Dercas N (2013). Simulation of soil water dynamics under surface drip irrigation from equidistant line sources. Water Resour. Manag..

[47] Arbat G, Puig-Bargués J, Duran-Ros M, Barragán J, Ramírez de Cartagena F (2013). Drip-Irriwater: Computer software to simulate soil wetting patterns under surface drip irrigation. Comput. Electron. Agric..

[48] Al-Ogaidi AAM, Aimrun W, Rowshon MK, Abdullah AF (2016). WPEDIS—Wetting pattern estimator under drip irrigation systems. Int. Conf. Agric. Food Eng..

[49] Karimi B, Mohammadi P, Sanikhani H, Salih SQ, Yaseen ZM (2020). Modeling wetted areas of moisture bulb for drip irrigation systems: An enhanced empirical model and artificial neural network. Comput. Electron. Agric..

[50] Richards, L. A. Capillary conduction of liquids through porous mediums. *Physics (College. Park. Md).* **1**, 318–333 (1931).

[51] Moncef H, Khemaies Z (2016). An analytical approach to predict the moistened bulb volume beneath a surface point source. Agric. Water Manag..

[52] Chen, J.-M., Tan, Y.-C. & Chen, Y.-Z. W. A study of the infiltration of trickle irrigation. in *7th International Micro Irrigation Congress* (PWTC Kuala Lumpur, 2006).

[53] Cook FJ, Thorburn PJ, Fitch P, Bristow KL (2003). WetUp: A software tool to display approximate wetting patterns from drippers. Irrig. Sci..

[54] Elmaloglou ST, Malamos N (2007). Estimation of width and depth of the wetted soil volume under a surface emitter, considering root water-uptake and evaporation. Water Resour. Manag..

[55] Ekhmaj AI, Amin MSM, Salim S, Zakaria A (2005). Wetted surface radius under point-source trickle irrigation in sandy soil. Int. Agric. Eng. J..

[56] Amin, M. S. M. & Ekhmaj, A. I. M. DIPAC—Drip irrigation water distribution pattern calculator. In *7th International Micro Irrigation Congress PWTC, Kuala Lumpur*. Vol. 1016. 503–513 (2006).

[57] Ekhmaj A, Abdulaziz A, Almdny A (2007). Artificial neural networks approach to estimate wetting pattern under point source trickle irrigation. Afr. Crop Sci. Conf. Proc..

[58] Kandelous MM, Šimůnek J, van Genuchten MT, Malek K (2011). Soil water content distributions between two emitters of a subsurface drip irrigation system. Soil Sci. Soc. Am. J..

[59] Zandi S, Nasab SB, Ainechee G (2008). Estimating soil moisture pattern in subsurface drip irrigation using dimensional analysis method. Iran. J. Agric. Sci..

[60] Arraes FDD, de Miranda JH, Duarte SN (2019). Modeling soil water redistribution under surface drip irrigation. Eng. Agríc..

[61] van Genuchten MT (1980). A closed-form equation for predicting the hydraulic conductivity of unsaturated soils. Soil Sci. Soc. Am. J..

[62] Van Genuchten, M. T. van Leij, F. J. & Yates, S. R. *The RETC Code for Quantifying the Hydraulic Functions of Unsaturated Soils*. (1991).

[63] Klute, A. Water retention: Laboratory methods. In *Methods of Soil Analysis*. 635–662. 10.2136/sssabookser5.1.2ed.c26 (2018).

[64] Klute, A. & Dirksen, C. Hydraulic conductivity and diffusivity: Laboratory methods. In *Methods of Soil Analysis: Part 1 Physical and Mineralogical Methods*. Vol. 5. 687–734 (Wiley Online Library, 2018).

[65] Mualem Y (1976). A new model for predicting the hydraulic conductivity of unsaturated porous media. Water Resour. Res..

[66] Lal R, Shukla MK (2004). Principles of Soil Physics.

[67] Rawls WJ, Brakensiek DL (1982). Estimating soil water retention from soil properties. J. Irrig. Drain. Div..

[68] Groenevelt PH, Grant CD (2004). A new model for the soil-water retention curve that solves the problem of residual water contents. Eur. J. Soil Sci..

[69] Michael AM (2009). Irrigation Theory And Practice.

[70] Kumar R, Nissa R, Kumar M (2018). Moisture dynamics and irrigation modelling in apple (*Malus domestica*) trees using CROPWAT model in temperate region of India. Indian J. Agric. Sci..

[71] Brandt A (1971). Infiltration from a trickle source: I. Mathematical models. Soil Sci. Soc. Am. J..

[72] Vishwakarma DK (2023). Forecasting of stage-discharge in a non-perennial river using machine learning with gamma test. Heliyon.

[73] Mirzania E, Vishwakarma DK, Bui Q-AT, Band SS, Dehghani R (2023). A novel hybrid AIG-SVR model for estimating daily reference evapotranspiration. Arab. J. Geosci..

[74] Elbeltagi A (2023). GLUE analysis of meteorological-based crop coefficient predictions to derive the explicit equation. Neural Comput. Appl..

[75] Samantaray S, Sahoo A, Satapathy DP (2022). Prediction of groundwater-level using novel SVM-ALO, SVM-FOA, and SVM-FFA algorithms at Purba-Medinipur, India. Arab. J. Geosci..

[76] Achite M (2023). Performance of machine learning techniques for meteorological drought forecasting in the Wadi Mina Basin, Algeria. Water.

[77] Elbeltagi A (2023). Prediction of meteorological drought and standardized precipitation index based on the random forest (RF), random tree (RT), and Gaussian process regression (GPR) models. Environ. Sci. Pollut. Res..

[78] Elbeltagi A (2022). Modelling daily reference evapotranspiration based on stacking hybridization of ANN with meta-heuristic algorithms under diverse agro-climatic conditions. Stoch. Environ. Res. Risk Assess..

[79] Vishwakarma DK (2022). Methods to estimate evapotranspiration in humid and subtropical climate conditions. Agric. Water Manag..

[80] Singh VK (2022). Novel genetic algorithm (GA) based hybrid machine learning-pedotransfer function (ML-PTF) for prediction of spatial pattern of saturated hydraulic conductivity. Eng. Appl. Comput. Fluid Mech..

[81] Ramsami P, Oree V (2015). A hybrid method for forecasting the energy output of photovoltaic systems. Energy Convers. Manag..

[82] Vishwakarma DK, Kumar R, Pandey K, Singh V, Kushwaha KS (2018). Modeling of rainfall and ground water fluctuation of Gonda District Uttar Pradesh, India. Int. J. Curr. Microbiol. Appl. Sci..

[83] Kim S, Kim H (2016). A new metric of absolute percentage error for intermittent demand forecasts. Int. J. Forecast..

[84] Malik A (2020). Modeling monthly pan evaporation process over the Indian central Himalayas: Application of multiple learning artificial intelligence model. Eng. Appl. Comput. Fluid Mech..

[85] Patel A (2023). Review of artificial intelligence and internet of things technologies in land and water management research during 1991–2021: A bibliometric analysis. Eng. Appl. Artif. Intell..

[86] Goodwin LD, Leech NL (2006). Understanding correlation: factors that affect the size of r. J. Exp. Educ..

[87] Asuero AG, Sayago A, González AG (2006). The correlation coefficient: An overview. Crit. Rev. Anal. Chem..

[88] Kushwaha NL (2022). Evaluation of data-driven hybrid machine learning algorithms for modelling daily reference evapotranspiration. Atmos. Ocean.

[89] Kushwaha N, Elbeltagi A, Mehan S, Malik A, Yousuf A (2022). Comparative study on morphometric analysis and RUSLE-based approaches for micro-watershed prioritization using remote sensing and GIS. Arab. J. Geosci..

[90] Markuna S (2023). Application of innovative machine learning techniques for long-term rainfall prediction. Pure Appl. Geophys..

[91] Almuhaylan MR (2020). Evaluating the impacts of pumping on aquifer depletion in arid regions using MODFLOW, ANFIS and ANN. Water.

[92] Gitau MW, Chaubey I (2010). Regionalization of SWAT model parameters for use in ungauged watersheds. Water.

[93] Provenzano G (2007). Using HYDRUS-2D simulation model to evaluate wetted soil volume in subsurface drip irrigation systems. J. Irrig. Drain. Eng..

[94] Cai Y (2022). Subsurface irrigation with ceramic emitters: Evaluating soil water effects under multiple precipitation scenarios. Agric. Water Manag..

[95] Sun G (2022). Optimizing irrigation and fertilization at various growth stages to improve mango yield, fruit quality and water-fertilizer use efficiency in xerothermic regions. Agric. Water Manag..

[96] Okasha AM (2022). Effects of irrigation method and water flow rate on irrigation performance, soil salinity, yield, and water productivity of cauliflower. Agriculture.

[97] Li Z, Zong R, Wang T, Wang Z, Zhang J (2021). Adapting root distribution and improving water use efficiency via drip irrigation in a jujube (*Zizyphus jujube* Mill.) orchard after long-term flood irrigation. Agriculture.

[98] Maurice B, Emile N, Charlotte U (2016). Assessment of wetting pattern and moisture distribution under point source drip irrigation in Nyagatare-Rwanda. Int. J. Innov. Sci. Res..

